# Integrated Plasmodium falciparum Transcriptomics Reveals Coordinated Stress Adaptation and Metabolic Remodeling in Severe Malarial Anemia

**DOI:** 10.21203/rs.3.rs-11245373/v1

**Published:** 2026-10-16

**Authors:** Douglas Perkins, Clinton Onyango, Shamim Osata, Sharley Wasena, Ivy Hurwitz, Philip Seidenberg, Collins Ouma, Samuel Anyona, Kristan Schneider, Benjamin McMahon, Qiuying Cheng

## Abstract

Malaria remains a leading cause of childhood morbidity and mortality in sub-Saharan Africa, with severe malarial anemia (SMA; hemoglobin (Hb) < 6.0 g/dL) representing one of the life-threatening complications of *Plasmodium falciparum* infection. While limited studies have examined host transcriptional responses in SMA, parasite transcriptional adaptations remain unexplored. Here, we performed parasite transcriptomic profiling in the same cohort of Kenyan children under five years of age in whom we previously characterized host transcriptional responses to SMA, including children presenting with SMA (n = 20) or non-SMA malaria (n = 40). Differential expression analysis identified 566 *P. falciparum* genes associated with SMA, predominantly involving up-regulated transcripts linked to proteostasis, translational regulation, stress-response pathways, and metabolic remodeling. Transcriptomic deconvolution indicated greater trophozoite and schizont representation in SMA, with reduced ring-stage and gametocyte signatures. Weighted gene co-expression network analysis and protein-protein interaction network analysis identified coordinated transcriptional programs enriched for proteasomal degradation, ubiquitin-mediated protein turnover, chaperone activity, RNA processing, and metabolic adaptation. XGBoost-SHAP modeling identified a small subset of parasite genes that captured much of the transcriptomic signal, classifying SMA from non-SMA malaria. Collectively, these findings define a distinct parasite transcriptional landscape in SMA characterized by coordinated stress adaptation and metabolic flexibility, highlighting proteostasis-associated pathways for further therapeutic investigation.

### INTRODUCTION

Malaria remains a major global health threat, with an estimated 282 million cases and 610,000 deaths in 2024, approximately 95% of which occurred in the WHO African region [1]. Children under five years of age accounted for 75% of malaria-related deaths in the region, primarily due to *Plasmodium falciparum* infections [1]. In holoendemic transmission regions such as Siaya County, western Kenya, severe malarial anemia (SMA, hemoglobin (Hb)<6.0 g/dL, any parasite density) [2] is among the most common and life-threatening manifestations of severe malaria in children under five [3]. The pathogenesis of SMA is multifactorial and involves hemolysis of infected and uninfected red blood cells (RBCs), splenic sequestration, dyserythropoiesis, and bone marrow suppression, often complicated by co-infections [4]. Despite ongoing malaria control efforts, SMA remains a major cause of pediatric morbidity and mortality in holoendemic regions [3].

Although host mechanisms contributing to SMA are beginning to be elucidated, considerably less is known about the transcriptional adaptations of *P. falciparum* to the severe inflammatory and nutrient-restricted host environment associated with SMA. Malaria-associated pathology develops during the intraerythrocytic developmental cycle of *P. falciparum*, a stage characterized by extensive and tightly regulated transcriptional activity [5-6]. Since *P. falciparum* mRNA contains poly(A) tails, next-generation RNA sequencing (RNA-Seq) of whole-blood samples enables simultaneous capture of parasite and host transcripts [7]. Peripheral blood transcriptomic analyses have therefore been used to investigate *in vivo* gene expression patterns during malaria infection [8-11]. For example, RNA sequencing of blood from Gambian children identified 236 parasite genes differentially expressed between severe malaria (SM) and uncomplicated malaria (UM), with enrichment of gene ontology (GO) terms related to RNA processing, hemoglobin catabolism, and protein transport [8]. Similarly, successive infections in Malian children showed altered parasite expression of phospholipase A2, Alba 2, and GAPDH, with transcriptional profiles differentiating early from later infections [9]. Additional studies of SM

(excluding SMA) linked parasite transcriptional alterations to biological processes including glycolysis, histone methylation, protein folding, and the tricarboxylic acid (TCA) cycle [10-11]. Collectively, these findings demonstrate the utility of transcriptomic profiling for parasite pathways associated with development, survival, virulence, metabolic adaptations, and immune evasion.

To extend these observations to SMA, we analyzed the *P. falciparum* transcriptome in a pediatric cohort previously used to characterize host transcriptional responses to SMA [2]. This complementary analysis defines parasite transcriptional programs associated with SMA, identifying altered expressions of genes involved in protein translation, proteostasis, RNA processing, stress responses, and metabolic adaptation. Network analyses further identified central parasite genes and pathways associated with SMA, while transcriptomic deconvolution revealed shifts in parasite developmental-stage composition. In addition, machine learning demonstrated that a relatively small subset of parasite genes classified SMA status. Together, these findings provide new insights into parasite adaptations associated with SMA and identify potential targets for future therapeutic investigation.

### RESULTS

#### ***Enrollment Characteristics of the Study Cohort***

Age- and sex-matched children without HIV-1 or bacteremia co-infections presenting with peripheral *P. falciparum* parasitemia (any density) were stratified at enrollment, prior to treatment interventions, into SMA (Hb<6.0 g/dL, n=20) and non-SMA (Hb≥6.0 g/dL, n=40). **Table 1** summarizes the demographic, clinical, and laboratory characteristics of the study participants.

Children with SMA had lower axillary temperatures ($p$=0.030), although both groups remained febrile, while glucose levels were comparable ($p$=0.709). Consistent with *a priori* Hb-based classification, children with SMA had lower hematocrit levels ($p$=3.539e-10) and red blood cell (RBC) counts ($p$=4.330e-10), accompanied by higher red cell distribution width ($p$=0.003) and mean corpuscular volume ($p$=0.003). White blood cell ($p$=0.001) and lymphocyte counts ($p$=1.140e-04) were also higher in SMA. No significant between-group differences were observed for mean corpuscular hemoglobin, platelet, monocyte, neutrophil, or granulocyte counts (all $p$>0.050). Sickle cell genotype distributions did not differ significantly between groups ($p$=0.079), although HbSS was more frequent among children with SMA.

#### ***Differential P. falciparum Gene Expression in SMA and non-SMA***

Differential gene expression analysis of 3,155 *P. falciparum* protein-coding genes identified 566 differentially expressed genes (DEGs) between the SMA and non-SMA groups (**Figure 1**). Of these, 499 genes were up-regulated, and 67 were down-regulated in SMA relative to non-SMA, demonstrating a predominance of increased parasite gene expression (499/566; 88%) in SMA.

#### ***Altered P. falciparum Developmental Stage Distributions in SMA***

Parasite densities did not differ significantly between SMA and non-SMA ($p$=0.520; **Figure 2a**). Transcriptomic deconvolution, however, revealed significant differences in the relative proportions of *P. falciparum* developmental stages between SMA and non-SMA (**Figure 2a and 2b**). Trophozoite and schizont proportions were higher in SMA ($p$=0.023 and $p$=0.001, respectively), whereas ring-stage parasites and female and male gametocytes were lower ($p$=0.002, $p$=0.020, and $p$=0.028, respectively; **Figure 2a and 2b**). Across all participants, female gametocyte proportions were significantly higher than male gametocytes. Females outranked males in 56 out of the 60 samples, with 4 samples resulting in a tie (Z=-6.509, $p$=7.550e-11; **Figure 2a**).

***WGCNA Identifies P. falciparum Transcriptional Modules Associated with SMA***

WCGNA of 3,155 annotated *P. falciparum* genes identified 12 co-expressed gene modules using a soft threshold power of 12.5 (**Figures 3a** and **3b**). Module eigengenes correlated with SMA status, identifying M9 ($r$=-0.37, $p$=0.038) and M10 ($r$=-0.34, $p$=0.028) as significantly associated modules (**Figure 3c**). Functional enrichment analysis of M9 revealed ATPase-coupled transmembrane transporter activity, proteasomal and ubiquitin-dependent protein catabolism, and enrichment of the proteasome and endopeptidase complexes (**Figure 4**). KEGG analysis further identified enrichment of proteasome-associated pathways. In contrast, M10 showed no significant functional enrichment. Hub-gene analysis identified ATP-dependent protease ATPase subunit ClpY (*Pf*ClpY) and heat shock protein 70 (*Pf*HSP70-3) as the central hub genes of modules M9 and M10, respectively (**Supplementary Table 1**).

***Protein-Protein Interaction Network Analysis Identifies Functional P. falciparum DEG Subnetworks in SMA***

To investigate functional relationships among the identified *P. falciparum* DEGs, PPI network analysis was performed. The global network comprised 542 nodes and 1,122 edges and showed significant PPI enrichment (*p*=4.06e-10), with 17 highly interconnected subnetworks identified by MCODE (**Figure 5; Table 2**). The global network hub node was PF3D7_1331700 (putative glutamine-tRNA ligase), which was also the hub of the highest-scoring subnetwork (cluster 1). Other prominent subnetworks were PF3D7_1474800 (putative proteasome subunit alpha type-1; cluster 2), PF3D7_0714000 (Histone H2B variant; cluster 3), PF3D7_0512600 (Ras-related protein Rab-1B; cluster 4), and PF3D7_0614400 (pre-mRNA-splicing factor CWF7, putative; cluster 5; **Table 2**). Of the WGCNA hub genes, *Pf*HSP70-3 was present in a densely connected PPI cluster enriched for cellular stress-response functions, whereas *Pf*ClpY was in the global network but not assigned to a significant MCODE cluster.

Functional enrichment analysis demonstrated that cluster 1 was enriched for ribosome biogenesis and protein synthesis, cluster 2 for proteasomal degradation and protein turnover, cluster 3 for chromatin remodeling and transcriptional regulation, cluster 4 for vesicle-mediated transport and intracellular trafficking, and cluster 5 for RNA splicing and mRNA processing pathways (**Figure 6a**). Enrichment profiles for clusters 6-10 and 11-17 are shown in **Figures 6b** and **6c**, respectively.

#### *XGBoost-SHAP Modeling Identifies P. falciparum Genes Associated with SMA Classification*

XGBoost modeling using parasite gene expression achieved moderate classification between SMA and non-SMA and outperformed clinical covariates alone (**Figure 7a**). Inclusion of clinical covariates with parasite gene expression features did not improve model performance [median AUC (IQR): covariates only=0.515 (0.313–0.625); genes only=0.719 (0.625–0.813); genes + covariates=0.719 (0.625–0.813)].

Cross-validated SHAP analysis identified a small group of parasite genes with consistently high contributions to SMA classification, led by PFKE01_130031100 (conserved *Plasmodium* protein, unknown function) and PFKE01_080024800 (perforin-like protein 5;

**Figure 7b**). Of the top-ranked features, PFKE01_080024800 consistently decreased predicted SMA risk, whereas most others showed context-dependent effects across individuals (**Figure 7b** and **7c**).

Incremental SHAP-based modeling showed that median ROC-AUC increased from 0.61 with the top-ranked gene alone to 0.72 with the top 20 genes, after which performance plateaued (**Figure 7d**). These findings indicate that a relatively small subset of *P. falciparum* genes captures predictive signal associated with SMA, with no incremental improvement from clinical covariates.

#### *Independent Assessment of P. falciparum Transcriptomic Signatures*

Validation was performed using an independent transcriptomic dataset from Gambian children with *P. falciparum* uncomplicated malaria (UM, n=21) or severe malaria (SM, n=25) reported by Lee *et al.* [8]. Differential expression analysis identified 428 parasite DEGs (332 up-regulated and 96 down-regulated) in SM relative to UM. Comparison with 566 parasite DEGs identified in the present study revealed 21 shared genes (**Figure 8a and b**). Fold changes among the shared genes showed a moderate inverse correlation between datasets ($r$=-0.48, $p$=0.029; **Figure 8c**). The limited gene-level concordance likely reflects differences in severe-malaria phenotypes, with the present study focused specifically on SMA and the validation cohort comprising cerebral malaria and/or hyperlactatemia without severe anemia.

Despite limited gene-level concordance, functional analyses revealed convergence between datasets. DEGs in the validation dataset were enriched for RNA metabolism, RNA processing, and spliceosomal functions, whereas the SMA dataset showed prominent enrichment of proteasomal and ubiquitin-dependent protein catabolism (**Supplementary Table 2**). PPI network analyses further identified shared functional themes involving RNA processing/splicing, ribosome/translation, and intracellular trafficking, while the SMA dataset additionally showed enrichment of protein degradation, protein folding/stress responses, and metabolic pathways (**Supplementary Tables 3 and 4; Supplementary Figure 1**).

WGCNA of the validation dataset identified 14 co-expression modules, none of which was significantly associated with disease status (**Supplementary Figure 2**). In contrast, XGBoost modeling demonstrated comparable classification performance, with parasite gene expression outperforming clinical covariates [median AUC (IQR): genes=0.750 (0.640–0.850); covariates=0.540 (0.425–0.660)]. Addition of clinical covariates did not improve performance (**Supplementary Figure 3**). Together, these analyses demonstrate limited gene-level concordance but functional convergence across distinct severe-malaria phenotypes, with parasite transcriptomic profiles retaining informative signals for disease classification.

### DISCUSSION

Severe malarial anemia remains one of the most life-threatening manifestations of pediatric *P. falciparum* malaria in holoendemic regions [3-4]. Although host mechanisms contributing to SMA during blood-stage *P. falciparum* infection, including parasite-induced hemolysis, immune-mediated RBC destruction, suppressed erythropoiesis, and dyserythropoiesis [4-5], have been investigated, parasite transcriptional adaptations remain undefined. Our previous analysis of the host peripheral blood transcriptome in this same pediatric cohort identified extensive immune, inflammatory, erythropoietic, and metabolic dysregulation associated with SMA [2]. The current complementary analysis reveals distinct parasite transcriptional signatures characterized by altered developmental-stage distributions and coordinated programs involving proteostasis, stress responses, and metabolic remodeling. Together, these findings begin to define both sides of the host-parasite transcriptional landscape associated with pediatric SMA.

Children with SMA exhibited the expected hematological features of severe anemia, including lower hematocrit and RBC counts and elevated RDW and MCV, consistent with altered erythropoiesis [12]. Elevated white blood cell and lymphocyte counts were also observed in SMA, consistent with heightened immune activation during severe disease [13]. Importantly, our previous transcriptomic analysis of these same children demonstrated extensive alterations in host immune, inflammatory, erythropoietic, and metabolic pathways associated with SMA [2]. Thus, the parasite transcriptional programs identified here occur within a profoundly altered host cellular and metabolic environment.

Despite differences in transcript abundance across most parasite genes, SMA was characterized by a predominance of up-regulated transcripts, suggesting selective activation of adaptive parasite pathways rather than global transcriptional disruption. Because developmental-stage composition can substantially influence bulk RNA-seq profiles, transcriptomic deconvolution was performed to assess parasite stage distributions.

Interpretation of circulating parasite stages is complicated by sequestration of mature trophozoites and schizonts [14] and tissue localization of developing gametocytes [15]. SMA was associated with reduced ring-stage and increased trophozoite and schizont proportions, indicating a shift toward more mature asexual stages. These findings are consistent with a transcriptomic meta-analysis reporting enrichment of mature trophozoites and reduced ring-stage proportions across distinct clinical malaria groups [16]. Based on the high metabolic activity of trophozoites, including extensive hemoglobin digestion and heme detoxification through hemozoin formation [17], increased representation of these stages may contribute to the transcriptional and metabolic differences observed in SMA. Together, these findings indicate altered intraerythrocytic developmental-stage composition in SMA, with greater representation of metabolically active mature asexual parasites. As such, some of the transcriptional differences associated with SMA may reflect altered developmental-stage composition, whereas the coordinated functional programs identified by subsequent network analyses suggest additional parasite responses associated with the SMA environment.

Both female and male gametocyte proportions were reduced in children with SMA, suggesting that severe anemia is associated with altered representation of transmission-stage parasites. Across the entire cohort, female gametocytes were more abundant than male gametocytes, consistent with the female-biased sex ratio characteristic of *P. falciparum* infections [18]. Male gametocytes have been reported to exhibit higher metabolic activity and protein expression than females [19]; however, whether the host environment associated with SMA differentially affects male and female gametocyte development remains unclear. Together with the increased representation of trophozoites and schizonts, the reduced gametocyte signatures suggest altered allocation between asexual and transmission-stage parasite populations in SMA.

WGCNA and PPI network analyses identified coordinated parasite transcriptional programs associated with proteostasis, protein turnover, stress adaptation, and metabolic regulation. A meta-analysis by Thomson-Luque *et. al*. similarly identified enrichment of proteasomal degradation, RNA processing, and translation-related pathways across *P. falciparum* transcriptomic datasets despite limited overlap among individual differentially expressed genes [16]. Thus, functional convergence may be more consistent than gene-level conservation across distinct clinical and epidemiological settings. In the present study, the M9 module was significantly associated with SMA and was enriched for ATP-dependent transport, ubiquitin-dependent protein catabolism, and proteasome-associated functions. These findings, together with the proteostasis-related signals identified by differential-expression and PPI analyses, suggest protein quality-control and turnover pathways as prominent components of the parasite transcriptional response associated with SMA.

Consistent with enriched proteostasis-related pathways, the M9 hub gene *Pf*ClpY was significantly up-regulated in SMA. *Pf*ClpY transcription begins during the trophozoite stage and peaks during late trophozoite and schizont development [20], suggesting that its elevated expression may reflect, at least in part, the greater representation of these mature asexual stages in SMA. *Pf*ClpY interacts with *Pf*ClpQ to form the ATP-dependent ClpQY protease complex involved in mitochondrial protein quality control [20]. Disruption of this interaction impairs mitochondrial membrane integrity and parasite growth [20]. The M10 hub gene *Pf*HSP70-3 was also up-regulated in SMA, a response demonstrated to protect the parasite against oxidative stress [21]. Additional proteostasis-related signals included HSP90-associated chaperone pathways and increased expression of the apicoplast chaperone ClpB1 and mitochondrial chaperone *Pf*HSP60 [22-23]. These parasite responses are important in the context of our previous transcriptomic analysis of the same children, demonstrating extensive host inflammatory and metabolic dysregulation associated with SMA [2]. Although the specific host factors driving these responses remain unclear, increased protease and chaperone expression suggest that parasites experience greater protein and organelle stress during SMA and may, in part, explain the low parasite densities in SMA observed in our cohort. Collectively, these findings support proteostasis and chaperone-mediated stress responses as central features of the parasite transcriptional landscape associated with SMA.

PPI network analysis further revealed coordinated changes in pathways involved in protein synthesis, including ribosome function, aminoacyl-tRNA synthesis, and RNA processing. These pathways are consistent with the increased translational demands of metabolically active asexual-stage parasites [24-25], which were higher in children with SMA. In addition, global PPI network hub PF3D7_1331700 (glutamine-tRNA ligase) and subnetwork hub PF3D7_1336900 (tryptophan–tRNA ligase) were both up-regulated in SMA. Because *P. falciparum* depends on host-derived amino acids (e.g., glutamine and tryptophan) for protein synthesis and intermediary metabolism [25-26], increased expression of these aminoacyl-tRNA synthetases may represent an adaptive response to altered nutrient availability in the severely anemic host. This interpretation is particularly relevant given the metabolic dysregulation identified in our previous host transcriptomic analysis of these same children [2]. In contrast, reduced expression of PF3D7_0409200 (SOF1, putative), a gene involved in ribosome biogenesis [27], suggests that not all components of the translational machinery are uniformly increased in SMA. Together, these findings indicate coordinated remodeling of parasite protein-synthesis pathways in the altered metabolic environment associated with SMA.

Proteasome-associated pathways also emerged prominently within the PPI subnetworks, providing additional evidence for altered protein quality control in parasites associated with SMA. Clusters enriched for ubiquitin-proteasome system (UPS) components included increased expression of genes involved in protein degradation and ubiquitin-mediated turnover, such as PF3D7_1353800 (proteasome subunit alpha type-1) and PF3D7_1363400 (polyubiquitin binding protein) [28-29]. Our previous investigation of the host UPS in Kenyan children with SMA identified differential expression of multiple human ubiquitination genes, with ubiquitin-proteasomal proteolysis emerging as the most prominently disrupted process [30]. Alterations in UPS-related pathways are, therefore, evident on both sides of the host-parasite interaction during SMA. Together with the chaperone and mitochondrial protein quality-control pathways identified here, these findings highlight proteostasis as a prominent feature of the molecular response associated with SMA. Based on the essential role of the UPS in parasite protein homeostasis and previous evidence supporting the *P. falciparum* proteasome as a drug target [28], these findings further support proteostasis pathways as candidates for therapeutic investigation.

Additional PPI subnetworks highlighted coordinated changes in chromatin regulation, DNA repair, and intracellular trafficking. Reduced expression of the histone H2B variant, PF3D7_1105100, suggests altered chromatin-associated regulation in SMA [31], whereas increased expression of the putative DNA repair-associated protein RAD14 (PF3D7_0710400) is consistent with increased DNA repair activity during asexual stage development [32]. Vesicle trafficking and retrograde transport pathways were also prominent, including increased expression of *Pf*Rab-1B and VPS26-associated processes [33-35]. These findings suggest that the parasite transcriptional response in SMA extends beyond proteostasis and involves pathways for genome maintenance and intracellular transport.

RNA-processing and post-translational regulatory pathways were also altered in parasites from children with SMA. Spliceosome-associated pathways were enriched, consistent with the importance of pre-mRNA processing in *P. falciparum*, in which more than half of genes contain introns [36]. Within the PPI network, the putative pre-mRNA processing factor CWF7 (PF3D7_0614400) was up-regulated, whereas splicing factor 3B subunit 6 (PF3D7_1224900, putative) was reduced [37-38]. This suggests remodeling rather than uniform activation of RNA-processing pathways. Of importance, RNA processing and spliceosome-associated pathways were also prominent in the independent severe-malaria transcriptomic dataset [8], indicating functional convergence across distinct severe-malaria phenotypes despite limited overlap at the individual-gene level. SUMOylation-associated pathways were also enriched, accompanied by increased expression of SUMO-activating enzyme subunit 2 (PF3D7_1237000), suggesting altered post-translational regulation in parasites associated with SMA [39].

Metabolic pathways identified through the PPI analyses indicate substantial remodeling of parasite metabolism in SMA. Enrichment of glycolytic pathways in the context of increased expression of ATP-dependent 6-phosphofructokinase is consistent with increased glycolytic demand during metabolically active asexual-stage development [40-41]. Lipid biosynthesis pathways were also enriched, as well as increased expression of cytidine diphosphate-diacylglycerol synthase, consistent with increased requirements for membrane synthesis during parasite growth and replication [42]. Mitochondrial metabolic pathways were also altered, including pathways for glutamine-dependent NAD(+) synthesis and reduced expression of *Pf*GDH1 [43-44]. These findings are particularly relevant in the context of our previous host transcriptomic analysis of the same children, which identified extensive alterations in host metabolic pathways associated with SMA [2]. Thus, coordinated metabolic changes occur in both the host and parasite, with parasite remodeling of glycolytic, lipid biosynthetic, and mitochondrial pathways occurring within a profoundly altered host metabolic environment. Collectively, these findings suggest that metabolic flexibility is a prominent feature of the parasite transcriptional response associated with SMA.

Machine-learning analysis provided an additional view of the parasite transcriptional features in children with SMA. Parasite gene expression classified SMA substantially better than the evaluated clinical covariates alone, with a median AUC of 0.719 compared with 0.515 for age, sex, and sickle-cell genotype. Moreover, adding these covariates to the parasite transcriptomic model did not improve classification. This suggests that parasite gene expression contained information associated with SMA that was not captured by these host characteristics. SHAP analysis further showed that this signal was concentrated within a relatively small number of genes. PFKE01_130031100, encoding a conserved *Plasmodium* protein of unknown function, and PFKE01_080024800, encoding perforin-like protein 5, were the two highest-ranking features. Although most highly ranked genes showed variable directional effects among individual samples, higher expression of perforin-like protein 5 consistently decreased the predicted probability of SMA. The function of the highest-ranked gene is unknown, and the biological significance of the perforin-like protein 5 association remains to be determined, prioritizing both as candidates for further investigation.

The incremental feature analysis further demonstrated that the SMA-associated transcriptomic signal was distributed across a small multigene signature rather than dominated by a single transcript. Classification performance increased from a median AUC of 0.61 using the highest-ranked gene alone to approximately 0.72 with the top 20 genes, after which additional features provided little improvement. Thus, although 3,155 parasite genes were evaluated, much of the classification signal was captured by a relatively small subset (20) of transcripts. This finding is consistent with the network analyses identifying coordinated parasite transcriptional programs rather than isolated gene-level responses. Nevertheless, the observed classification performance was moderate and should not be interpreted as establishing a clinical diagnostic or predictive model. The relatively small cohort and cross-sectional design also preclude determination of whether these transcriptional features contribute to SMA pathogenesis or develop in response to the altered host environment associated with severe anemia. Developmental-stage differences between SMA and non-SMA parasites may also contribute to the classification signal. The machine-learning results therefore identify a focused set of parasite genes and transcriptional patterns for functional investigation and validation in larger independent cohorts.

Comparison with the independent Gambian severe-malaria dataset [8] revealed limited concordance at the individual-gene level but substantially greater convergence at the functional level. Of the 566 DEGs associated with SMA and 428 identified in the independent severe-malaria dataset, only 21 genes overlapped, and their fold changes were moderately inversely correlated but significant. This limited gene-level agreement is not unexpected given the distinct clinical phenotypes: the present study specifically compared SMA with non-SMA malaria, whereas the Gambian severe-malaria group was children with cerebral malaria and/or hyperlactatemia without severe anemia. Despite these differences, both datasets converged on parasite processes involving RNA processing and splicing, ribosome and translation functions, and intracellular trafficking. In contrast, the SMA dataset showed more prominent protein degradation and turnover, protein folding and stress responses, and metabolic pathways. This pattern is consistent with the meta-analysis of Thomson-Luque *et al.*, in which individual parasite transcriptional signatures varied across malaria studies while broader biological programs showed greater convergence [16]. The independent dataset therefore supports conservation of functional parasite responses across distinct severe-malaria phenotypes, while the prominent proteostasis, stress-response, and metabolic signals identified here warrant further investigation for their relationship with SMA.

The machine-learning results provide additional concordance between cohorts. Parasite gene-expression profiles classified disease status with similar moderate performance in the SMA cohort (median AUC, 0.719) and the independent severe-malaria cohort (median AUC, 0.750). Moreover, the evaluated clinical covariates performed substantially less well in both datasets and did not improve the transcriptome-based models. Thus, although the specific genes associated with disease differed between cohorts, parasite transcriptional profiles retained classification-relevant information across distinct manifestations of severe malaria. These parasite responses also occurred within a host environment that we previously showed, using the same pediatric SMA cohort, to be characterized by extensive immune, inflammatory, erythropoietic, and metabolic dysregulation [2]. Considered together, the host and parasite transcriptomes provide complementary perspectives on the molecular interplay associated with SMA. However, direct functional relationships between specific host and parasite pathways remain to be established.

Overall, complementary transcriptomic approaches converge on a parasite landscape in SMA characterized by altered developmental-stage composition and coordinated changes in proteostasis, protein turnover, translational regulation, stress responses, and metabolism. Proteasome- and ubiquitin-associated pathways were particularly pronounced across differential-expression, WGCNA, enrichment, and PPI analyses, while glycolytic, lipid biosynthetic, amino-acid utilization, and mitochondrial pathways indicate extensive metabolic remodeling. These parasite responses occur alongside the host transcriptional dysregulation previously identified in the same children and, at the functional level, share core features with an independent severe-malaria parasite transcriptome. Functional studies will be required to determine which parasite responses contribute to SMA pathogenesis and which primarily reflect adaptation to the altered host environment. The convergence of proteostasis, stress-response, and metabolic pathways identifies focused processes for mechanistic investigation and supports further evaluation of parasite proteostasis pathways as candidates for therapeutic investigation in severe pediatric malaria.

### METHODS

#### *Study Area and Participants*

The study was conducted between March 2017 and September 2020 at Siaya County Referral Hospital (SCRH) in western Kenya, a *P. falciparum* holoendemic transmission region with high rates of malaria-related morbidity and mortality among children under five years of age [3, 45]. Children were eligible for enrollment if they were 1-59 months of age, had *P. falciparum* parasitemia of any density, presented with an axillary temperature ≥37.5 °C, resided within 25 km of SCRH, had written informed consent provided by a parent or legal guardian, and were willing to return for a day-14 follow-up visit. Children were excluded if they had been previously hospitalized, experienced a malaria episode within the preceding month, or presented with clinical signs consistent with cerebral malaria.

A total of 577 children were enrolled. At enrollment, demographic and clinical information were collected, a physical examination was performed, and peripheral blood was obtained before initiation of antimalarial treatment. Children were classified as having severe malarial anemia (SMA; Hb<6.0 g/dL; *n*=124) or non-SMA malaria (Hb≥6.0 g/dL; *n*=453) according to established definitions [2].

For transcriptomic analyses, children with SMA and non-SMA malaria were selected to achieve comparable age and sex distributions between groups, and participants with HIV-1 infection and/or bacteremia were excluded. Whole-blood RNA-seq was performed on samples collected at enrollment, before treatment, from 66 children (SMA, n=25; non-SMA, n=41). Pre- and post-HIV test counseling was provided to parents or legal guardians of all participants. All children received treatment according to Ministry of Health Kenya guidelines.

#### *Ethics Statement*

The study was approved by the University of New Mexico Health Sciences Center Human Research Review Committee (protocol no. 17-445) and the Maseno University Scientific and Ethics Review Committee (protocol no. MSU/DRPI/MUERC/00510/18). Written informed consent was provided by the parents or legal guardians of the study participants.

#### ***Sample Collection and Laboratory Procedures***

Peripheral blood (3-4 mL) was collected by venipuncture from each study participant on enrollment (day 0) prior to initiation of antimalarial therapy. Thick and thin Giemsa-stained blood smears were prepared and examined microscopically under 100x oil immersion objective to determine *Plasmodium* infection and density [45].

Hb concentrations and complete blood counts (CBCs) were measured using the Beckman Coulter ACT diff2™ hematology analyzer (Beckman-Coulter Corporation, Miami, FL, USA). HIV-1 infection, bacteremia, and hemoglobin variants were evaluated for all children to identify potential contributors to severe anemia and to determine eligibility for the transcriptomic analyses. HIV-1 exposure was assessed serologically using the Uni-Gold™ HIV test (Trinity Biotech USA Inc., Jamestown, NY, USA) and Determine™ HIV-1/2 test (Abbott Diagnostics Scarborough, Inc., Scarborough, ME, USA). Samples reactive by either rapid test were subsequently evaluated for HIV-1 infection by nested proviral DNA PCR, as previously described [46].

To assess bacteremia, ~1.0 mL of venipuncture blood was inoculated into the automated BACTEC 9050 system (Becton, Dickinson and Company, Franklin Lakes, NJ, USA). Positive cultures were evaluated by Gram staining and subcultured on blood agar, chocolate agar, or MacConkey agar [47]. Bacterial isolates were identified using API biochemical galleries (bioMérieux Inc., Durham, NC, USA) [47]. Major hemoglobin variants (i.e., HbA, HbS, HbC, and HbF) were characterized by cellulose acetate electrophoresis (Helena Bio-Sciences, Beaumont, TX, USA).

For transcriptomic analyses, an aliquot of peripheral blood (~500 μL) was mixed with an equal volume of Trizol® reagent (Thermo Fisher Scientific Inc., Waltham, MA, USA) and stored at -80 °C until RNA isolation.

***RNA Isolation***

Total RNA was isolated from peripheral blood preserved in TRIzol® reagent using the E.Z.N.A.® Total RNA Kit I (Omega Bio-Tek Inc., Norcross, GA, USA). To remove contaminating genomic DNA, RNA samples were treated with RNase-free DNase I (New England Biolabs, Ipswich, MA, USA). RNA concentration was measured using a NanoDrop 2000 Spectrophotometer (Thermo Fisher Scientific), and RNA integrity was assessed using an Agilent 2100 Bioanalyzer (Agilent Technologies, Santa Clara, CA, USA).

***Library Construction for RNA Sequencing***

A total of 1 μg of RNA with an RNA integrity number (RIN) >8, following globin mRNA depletion, was used for sequencing library preparation. Libraries were constructed using NEBNext® Ultra™ RNA Library Prep Kit for Illumina® (New England BioLabs Inc.). Briefly, mRNA was enriched using poly-T oligo-attached magnetic beads and fragmented using divalent cations at elevated temperature in 2X NEBNext First Strand Synthesis Reaction Buffer. First-strand cDNA was synthesized using random hexamer primers and M-MuLV Reverse Transcriptase (RNase H-), followed by second-strand cDNA synthesis using DNA Polymerase I and RNase H. The resulting cDNA fragments were ligated to NEBNext adaptors and amplified by PCR. Final PCR products were purified, and library quality was assessed using an Agilent 2100 Bioanalyzer (Agilent Technologies).

***RNA Sequencing and Data Pre-processing***

Clustering generation of index-coded samples was performed using the cBot Cluster Generation System with the PE Cluster Kit cBot-HS (Illumina Inc.). Paired-end sequencing was conducted on an Illumina® NovaSeq S4 platform (Illumina Inc.), generating >20 million high-quality, mappable reads per sample.

Quality control and pre-processing of raw FASTQ reads were performed using fastp [48]. Adapter-contaminated reads, reads containing poly-N sequences, and low-quality reads and bases were removed to generate clean reads for downstream analyses. All subsequent analyses were performed using the resulting filtered reads.

#### *Mapping to the Plasmodium Reference Genome and Gene Quantification*

To complement our previously published host transcriptomic analysis generated from the same RNA sequencing libraries [2], clean sequencing reads were analyzed for parasite gene expression by alignment to the *P. falciparum* Kenyan isolate (PFKE01 release 51) reference genome, obtained from PlasmoDB [49]. Clean RNA-seq reads were aligned to the PFKE01 reference genome using Bowtie 2 [50], and gene-level read counts were quantified using HTSeq v0.6.1 [51]. Samples failing quality control criteria and/or containing <10,000 reads mapped to the PFKE01 genome were excluded from downstream analyses. This resulted in the removal of six samples (5 SMA and 1 non-SMA). The final dataset included 60 samples comprising 20 SMA and 40 non-SMA cases. Gene expression counts were normalized to transcripts per million (TPM) to account for transcript length and sequencing depth.

#### *Data Analysis:*

#### *Demographic, Clinical, and Laboratory Characteristics of the Study Participants*

Demographic, clinical, and laboratory characteristics of the study participants at enrollment were analyzed using IBM SPSS Statistics version 23.0 (IBM Corp., Armonk, NY, USA). Comparisons between the SMA and non-SMA groups were performed using Fisher's exact test for categorical variables and the Mann-Whitney *U* test for continuous variables. Statistical significance was defined as $p \leq 0.050$. Continuous variables are presented as medians and interquartile ranges (IQRs), and categorical variables as counts and percentages.

#### ***Parasite Differential Gene Expression*** **Analysis**

A total of 3,155 annotated *P. falciparum* protein-coding genes were analyzed for differential expression between the SMA and non-SMA groups. Differential gene expression analysis was performed using the Welch-corrected *t*-test to account for unequal variances and differences in sample sizes between groups. DEGs were defined as genes exhibiting significantly different mean expression levels between the SMA and non-SMA ($p \leq 0.050$). The distribution of differentially expressed protein-coding genes was visualized using a volcano plot generated in GraphPad Prism version 9.0 (GraphPad Software, Inc., Boston, MA, USA). To ensure compatibility with downstream analytical platforms requiring *P. falciparum* 3D7 gene annotations, PFKE01 gene identifiers for DEGs were mapped to their corresponding PF*3D7* orthologs using PlasmoDB and NCBI.

#### ***P. falciparum Gene Expression Deconvolution Analysis***

Gene expression deconvolution was performed using CIBERSORTx [52] to estimate the relative proportions of *P. falciparum* developmental stages within each sample. Reference files, including the signature matrix and single-cell RNA-seq expression matrix, were obtained from a previously published single-cell RNA-seq study [53]. Batch correction was performed using S-mode to account for technical differences between the single-cell reference and bulk RNA-seq datasets, with 1000 permutations used for statistical inference.

Estimated proportions of *P. falciparum* developmental stages were visualized using GraphPad Prism version 9.0 (GraphPad Software, Inc.). Relative stage proportions were compared between the SMA (n=20) and non-SMA (n=40) groups using the Mann-Whitney *U*

test.  Paired differences between male and female gametocyte proportions across all samples (n=60) were evaluated using the Wilcoxon signed-rank test.  Statistical analyses were performed using IBM SPSS Statistics version 23.0 (IBM Corp.).

***Weighted Gene Co-expression Network Analysis (WGCNA)***

To identify *P. falciparum* gene co-expression patterns associated with SMA, WGCNA was performed in R (version 4.4.1) [54] using TPM-normalized expression data of 3,155 parasite genes.  Gene expression data were log-transformed, and genes lacking expression variability were excluded prior to analysis.  A soft-thresholding power of 12.5 was selected based on the scale-free topology criterion ($R^2$>0.8).  Gene co-expression networks were constructed using the WGCNA R package [54], with a minimum module size of 75 genes.  Identified gene modules were assigned unique colors for visualization and downstream analysis.

For each module, the module eigengene (ME), representing the first principal component of module gene expression within the module, was calculated and correlated with SMA status (coded as 0 and 1) using weighted Pearson correlation analysis.  Resulting *p*-values were adjusted for multiple testing using the Benjamini-Hochberg method.  For modules significantly associated with SMA status, module membership (MM) and intramodular connectivity (kWithin) were calculated, and genes with the highest kWithin were identified as hub genes.

Functional enrichment analysis was subsequently performed on genes within SMA-associated modules using the gprofiler2 R package [55].  PFKE01 gene identifiers were mapped to their corresponding PF3D7 orthologs prior to enrichment analysis.  Gene Ontology (GO) and Kyoto Encyclopedia of Genes and Genomes (KEGG) enrichment analyses were performed using the hypergeometric test, with correction for multiple testing using the gProfiler

Set Counts and Sizes method. Enrichment results were visualized using the Scientific and Research Plot (SRplot) tool [56].

#### *Protein-Protein Interaction (PPI) Network Analysis of Proteins Encoded by P. falciparum DEGs*

A protein-protein interaction (PPI) network of proteins encoded by *P. falciparum* DEGs was generated using STRING Version 12.0 (https://string-db.org/), with a minimum interaction confidence score of 0.700 (high confidence). The resulting network was imported into and visualized using Cytoscape [57]. Highly interconnected sub-networks (clusters) within the global PPI network were identified using the Molecular Complex Detection (MCODE) plugin (version 2.0.3) [58]. Clusters with MCODE scores >4 were retained and ranked according to their MCODE scores. Functional enrichment analysis of genes within each retained cluster was performed using the stringApp in Cytoscape, and enrichment results were visualized using SRplot [56].

Hub nodes within the global PPI network and individual MCODE clusters were identified using the CytoHubba plugin (version 0.1) in Cytoscape. Nodes were ranked using 11 topological algorithms: Degree, Edge Percolated Component, Maximum Neighborhood Component, Density of Maximum Neighborhood Component, Maximal Clique Centrality, and six centrality-based measures (Bottleneck, Eccentricity, Closeness, Radiality, Betweenness, and Stress). For each node, 10 values from each algorithm were averaged, and the resulting 11 mean scores summed to generate an aggregate consensus score. Nodes were ranked by this score, with the highest-scoring nodes identified as global and cluster-specific hubs [59].

#### *Machine Learning Classification of SMA Using P. falciparum Gene Expression Profiles*

To evaluate the ability of *P. falciparum* gene expression profiles to distinguish SMA status, classification was performed using XGBoost Python Package (version 1.7.6) with repeated stratified cross-validation (CV) [60]. Three models were evaluated using (i) 3,155 parasite genes, (ii) age, sex, and sickle-cell genotype, or (iii) parasite genes combined with these covariates. Sickle-cell genotype was one-hot encoded. Models used 100 estimators, a maximum tree depth of 3, and a learning rate of 0.1, were evaluated using stratified 5-fold cross-validation repeated 200 times with shuffling. Performance was assessed on held-out test folds using the area under the receiver operating characteristic curve (ROC-AUC) and summarized as the median and interquartile range (IQR) across folds and repeats.

Feature importance was evaluated using SHAP (SHapley Additive exPlanations; version 0.49.1) [61]. Interventional TreeSHAP values were calculated on held-out test samples using models trained within each CV fold. Feature importance was summarized using median absolute SHAP values, with median signed SHAP values used to assess the direction of contributions to predicted SMA probability. To determine the number of features required for maximal classification performance, leakage-free incremental top-k modeling (k=1-50) was performed. Within each training fold, features were ranked by mean absolute SHAP value and separate XGBoost models containing the top-k features were evaluated on the corresponding held-out test fold. Performance was summarized as the median ROC-AUC across all folds and repeats.

**Independent Assessment of *P. falciparum* Transcriptomic Signatures**

To assess whether parasite transcriptomic patterns identified in SMA were also present in an independent severe-malaria cohort, we searched for publicly available *P. falciparum* transcriptomic datasets with comparable clinical phenotypes. Since no independent dataset directly comparing SMA with non-SMA was identified, the dataset reported by *Lee et al*. [8] (accession no. E-MTAB-6413) was selected as the closest available severe malaria comparison. The dataset comprised whole-blood transcriptomic profiles from an independent cohort of Gambian children aged 1.5 to 15 years with uncomplicated *P. falciparum* malaria (UM, n=21) or severe malaria (SM, n=25). The SM group included children with cerebral malaria and/or hyperlactatemia but no cases of severe anemia, whereas the UM group included children with *P. falciparum* parasitemia without severe malaria manifestations.

Raw sequencing reads from the independent dataset were downloaded and processed using the same bioinformatic and analytical workflows applied to the present study. Sequencing reads were aligned to the PFKE01 reference genome, followed by differential-expression, functional-enrichment, PPI-network, and XGBoost analyses using the procedures and criteria described above. WGCNA was performed using the same workflow, with a dataset-specific soft-thresholding power of 5 selected to achieve scale-free topology ($R^2$>0.8). Because the clinical phenotypes differed between the datasets (SMA vs. non-SMA and SM vs. UM), comparisons were interpreted as an independent assessment of gene-level and functional patterns rather than direct replication of SMA-specific molecular features.

***Data Availability***

The raw and processed RNA-seq data generated in this study are publicly available through the NCBI Gene Expression Omnibus (GEO) under accession number GSE316842. The publicly available raw sequencing data used for the independent severe-malaria analysis are available under accession number E-MTAB-6413 [8].

**ACKNOWLEDGEMENTS**

The authors thank the University of New Mexico-Kenya team for their contributions to the study: Nicholas O. Ondiek, Anne A. Ong'ondo, Everlyne A. Modi, Joan L. A. Ochieng, Duncan O. Njega, Joseph Oduor, Moses Ebungure, Moses Lokorkeju, Rodney B. Mongare, and Vincent Omanje. We thank the children who participated in the study and their parents and guardians.

FUNDING

This work was supported by the National Institutes of Health (NIH) through research grants R01AI130473 (D.J.P. and S.B.A.), R01AI51305 (D.J.P.), and the NIH Fogarty International Center through grants K43TW011581 (S.B.A.), D43TW05884 (D.J.P. and C.O.), and D43TW010543 (D.J.P).

**COMPETING INTERESTS**

The authors declare no competing interests.

**Contributor Information**

**Authors and Affiliations**

**Department of Internal Medicine, Center for Global Health, University of New Mexico, Albuquerque, NM, 87131-0001, USA**
Clinton O. Onyango, Qiuying Cheng, Ivy Hurwitz, Kristan A. Schneider & Douglas J. Perkins

**Department of Biomedical Sciences and Technology, School of Public Health and Community Development, Maseno University, Maseno, 40105, Kenya**
Clinton O. Onyango, Shamim W. Osata, Sharley A. Wasena, & Collins Ouma

**Department of Emergency Medicine, School of Medicine, University of New Mexico, Albuquerque, NM, 87131-0001, USA**
Philip D. Seidenberg

**University of New Mexico-Kenya Global Health Programs, Kisumu and Siaya, 40100, Kenya**
Shamim W. Osata, Sharley A. Wasena, Collins Ouma, Samuel B. Anyona, & Douglas J. Perkins

**Department of Medical Biochemistry, School of Medicine, Maseno University, Maseno, 40105, Kenya**
Samuel B. Anyona

**Department of Internal Medicine, Division of Translational Informatics, University of New Mexico, Albuquerque, NM, 87131-0001, USA**
Kristan A. Schneider

**Theoretical Biology and Biophysics Group, Theoretical Division, Los Alamos National Laboratory, Los Alamos, NM, 87545, USA**

Benjamin H. McMahon

**Contributions**

COO: Conducted experiments, data analysis, and manuscript writing. SWO: Data analysis and manuscript review and editing. SAW: Data analysis and manuscript review and editing. IH: Project supervision, technical support, manuscript review and editing. PDS: Manuscript review and editing. CO: Project supervision, manuscript review and editing. SBA: Project supervision, conducted experiments, manuscript review and editing. KAS: Data analysis and manuscript review and editing. BHM: Data analysis and manuscript review and editing. QC: Project supervision, technical support, data analysis, and manuscript review and editing. DJP: Designed the study, project supervision, data analysis, and co-wrote the manuscript.

**Table 1. Demographic, Clinical, and Laboratory Characteristics of Study Participants at Enrollment**

| Characteristics | Non-SMA (Hb≥6.0 g/dL) | SMA (Hb<6.0 g/dL) | *p*-value |
|---|---|---|---|
| No. of participants (n=60) | 40 | 20 | |
| Gender, n (%) | | | |
| Male | 19 (47.5) | 9 (45.0) | 1.000[a] |
| Female | 21 (52.5) | 11 (55.0) | |
| Age, months | 23.5 (22.3) | 22.5 (27.5) | 0.724[b] |
| Glucose, mmol/L | 5.1 (2.4) | 4.7 (1.9) | 0.709[b] |
| Admission temperature, °C | 38.0 (1.1) | 37.7 (0.9) | **0.030**[b] |
| **Hematological Parameters** | | | |
| Hemoglobin, g/dL | 9.9 (1.4) | 4.6 (1.0) | NA |
| Hematocrit, % | 30.0 (5.4) | 14.7 (2.7) | **3.539e-10**[b] |
| Red blood cells, × $10^6$/µL | 4.3 (0.9) | 1.9 (0.8) | **4.330e-10**[b] |
| Red cell distribution width, % | 18.5 (2.8) | 21.6 (7.9) | **0.003**[b] |
| Mean corpuscular volume, fL | 69.5 (8.4) | 80.1 (28.8) | **0.003**[b] |
| Mean corpuscular haemoglobin, pg | 22.7 (4.2) | 25.9 (9.2) | 0.051[b] |
| Platelets, ×$10^3$/µL | 144.0 (99.3) | 100.0 (159.5) | 0.981[b] |
| Platelet distribution width, % | 16.5 (1.2) | 17.2 (1.0) | 0.441[b] |
| Mean platelet volume, fL | 8.3 (2.5) | 8.0 (2.4) | 0.190[b] |
| White blood cells, ×$10^3$/µL | 11.0 (6.8) | 21.4 (10.1) | **0.001**[b] |
| Lymphocytes, ×$10^3$/µL | 3.7 (1.8) | 7.5 (10.1) | **1.140e-04**[b] |
| Monocytes, ×$10^3$/µL | 1.2 (1.3) | 1.7 (1.7) | 0.063[b] |
| Neutrophils, ×$10^3$/µL | 5.3 (3.4) | 5.2 (8.1) | 0.780[b] |
| Granulocytes, ×$10^3$/µL | 6.7 (3.0) | 8.6 (5.1) | 0.540[b] |
| **Genetic Variants** | | | |
| Sickle cell genotypes, n (%) | | | |
| HbAA | 35 (87.5) | 14 (70.0) | |
| HbAS | 4 (10.0) | 2 (10.0) | 0.079[a] |
| HbSS | 1(2.5) | 4 (20.0) | |

Data presented is the median (interquartile range; IQR) unless otherwise indicated. Children presenting with malaria at SCRH were stratified by hemoglobin concentration into non-severe malarial anemia (non-SMA; Hb ≥ 6.0 g/dL, n = 40) and severe malarial anemia (SMA; Hb < 6.0 g/dL, n = 20). [a]Fisher's exact test with exact *p*-values for homogeneity and [b]Mann-Whitney U test were used to compare the study groups. Statistical significance was defined as *p*≤0.050. Significant *p*-values are indicated in bold.

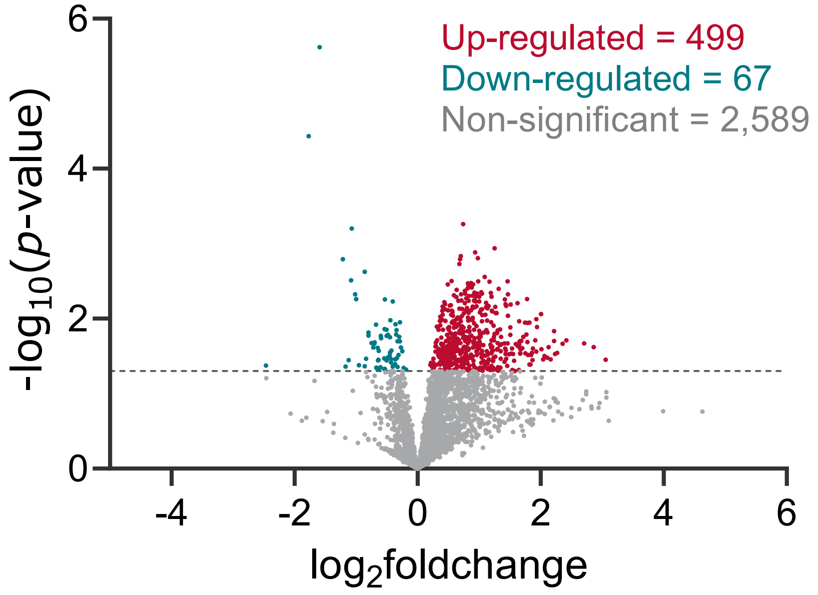

**Figure 1**. **Differential Expression of *P. falciparum* mRNA Genes in SMA and non-SMA.** Volcano plot showing differential expression of 3,155 annotated *P. falciparum* genes between SMA and non-SMA groups. A total of 499 genes were up-regulated (red) and 67 down-regulated (turquoise) in SMA relative to non-SMA. The x-axis represents $\log_2$foldchange, and the y-axis represents $-\log_{10}$ (*p*-values). The horizontal dashed line at 1.301 [i.e., $\log_{10}$ (*p*-value)] indicates the significance threshold at 0.050.

a

| | Non-SMA (Hb≥6.0 g/dL) | SMA (Hb<6.0 g/dL) | *p*-value |
|---|---|---|---|
| Parasite density, MPS/µL | 59,417 (81,968) | 29,036 (80,7347) | 0.520[a] |
| *P. falciparum* stage | | | |
| Ring | 0.448 (0.372) | 0.299 (0.382) | **0.002[a]** |
| Trophozoite | 0.294 (0.587) | 0.561 (0.523) | **0.023[a]** |
| Schizont | 0.000 (0.002) | 0.033 (0.162) | **0.001[a]** |
| Female gametocyte | 0.120 (0.125) | 0.080 (0.110) | **0.020[a]** |
| Male gametocyte | 0.073 (0.076) | 0.021 (0.068) | **0.028[a]** |
| ***P. falciparum*** **gametocytes** | | | |
| Sex | Female | Male | |
| | 0.110 (0.121) | 0.059 (0.077) | **7.55e-11[b]** |

b

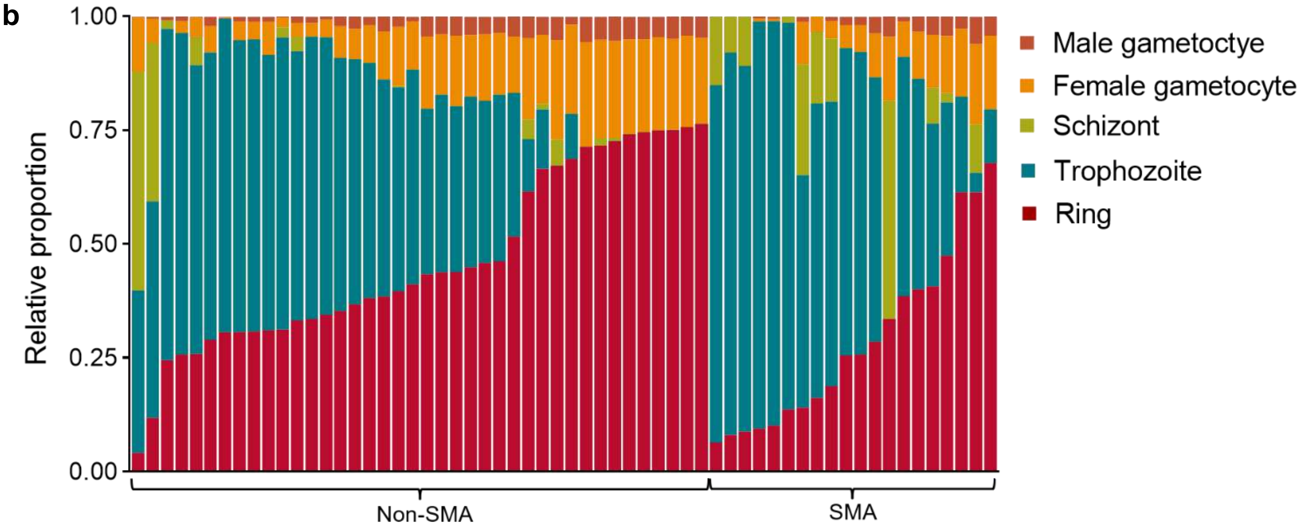

**Figure 2. Transcriptomic Deconvolution Reveals Altered *P. falciparum* Developmental Stage Distributions in SMA**. (**a**) Relative proportions of *P. falciparum* developmental stages in non-SMA (n=40) and SMA (n=20), together with relative proportions of female and male gametocytes across all samples (n=60). Data are presented as median (interquartile range; IQR). [a]Mann-Whitney U test and [a]Wilcoxon signed-rank test. Statistical significance was defined as *p*≤0.050, and significant *p*-values are shown in bold. (**b**) Relative proportions of inferred *P. falciparum* developmental stages estimated from parasite transcriptomic profiles.

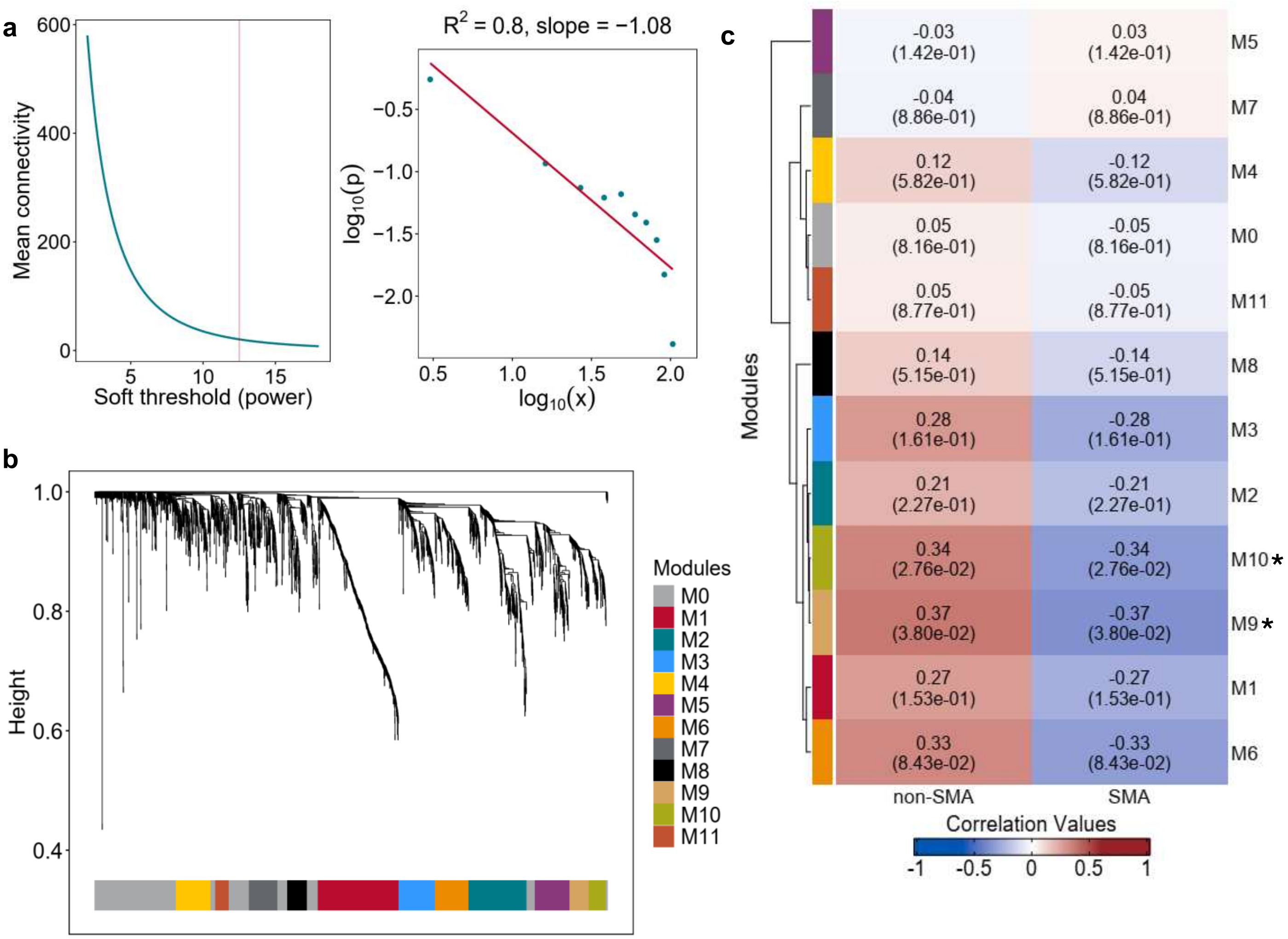

**Figure 3. WGCNA Identifies Co-expressed *P. falciparum* Gene Modules Associated with SMA.** (**a**) Determination of soft-thresholding power in the WGCNA. Mean connectivity plot showing mean connectivity analysis of various soft-thresholding powers (β) and a power-law plot showing a negative correlation between connectivity (k) and probability distribution of connectivity (p) (correlation coefficient 0.80). (**b**) Gene dendrogram and module colors. Hierarchical clustering tree represents the clustering of genes based on the TOM-based dissimilarity matrix (1-TOM) as the distance measure. The branches represent clusters of highly connected genes, and each vertical line represents a single gene. The colors in the horizontal bar represent the 12 gene co-expression modules identified using the dynamic tree cutting algorithm. (**c**) Heatmap of module-clinical trait relationships. The heatmap shows the correlation between WGCNA module eigengenes and SMA status. Each cell contains the correlation coefficient and Benjamini-Hochberg adjusted *p*-value in parentheses. Rows and columns represent modules and clinical features, respectively. The color intensity represents the strength of the correlation. Red indicates a positive correlation, while blue represents a negative correlation. *Indicates a significant module-trait relationship.

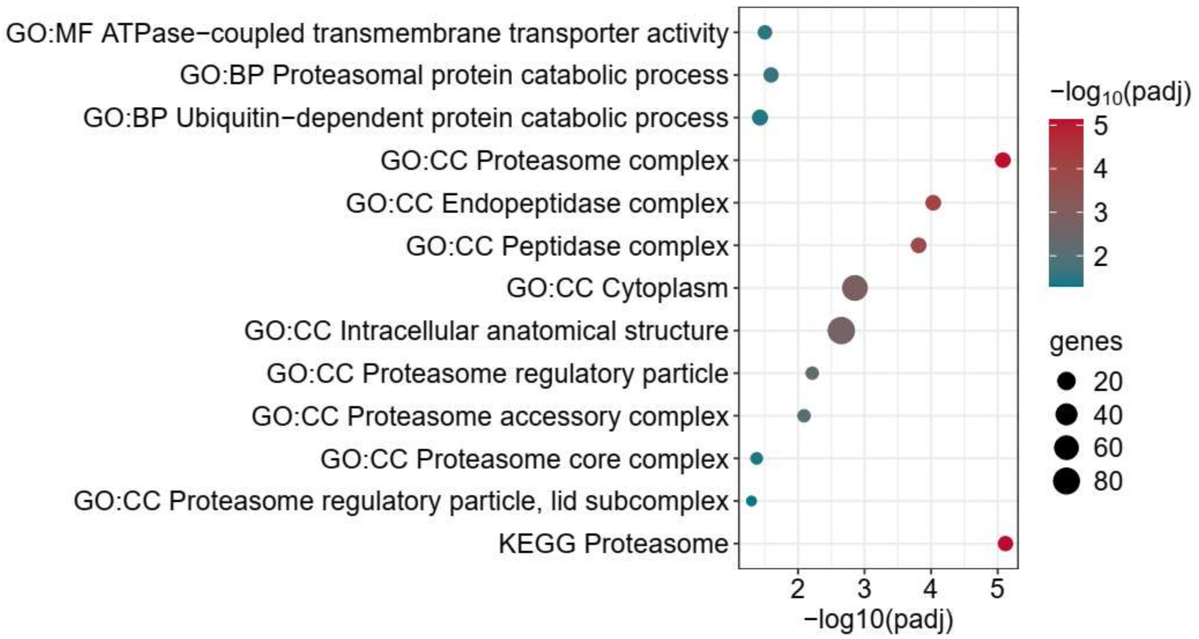

**Figure 4. Functional Enrichment Analysis of WGCNA Module M9.** Functional enrichment plots show significant functional enrichment terms associated with co-expressed genes within the WGCNA modules. The x-axis represents the -$\log_{10}$ (FDR-adjusted *p*-value), and the y-axis shows enriched functional terms. Terms are grouped by GO (Gene Ontology) [MF (Molecular Function), CC (Cellular Component), and BP (Biological Process)] and KEGG (Kyoto Encyclopedia of Genes and Genomes) pathways. Enrichment terms with *padj*-values ≤0.050 were considered significant.

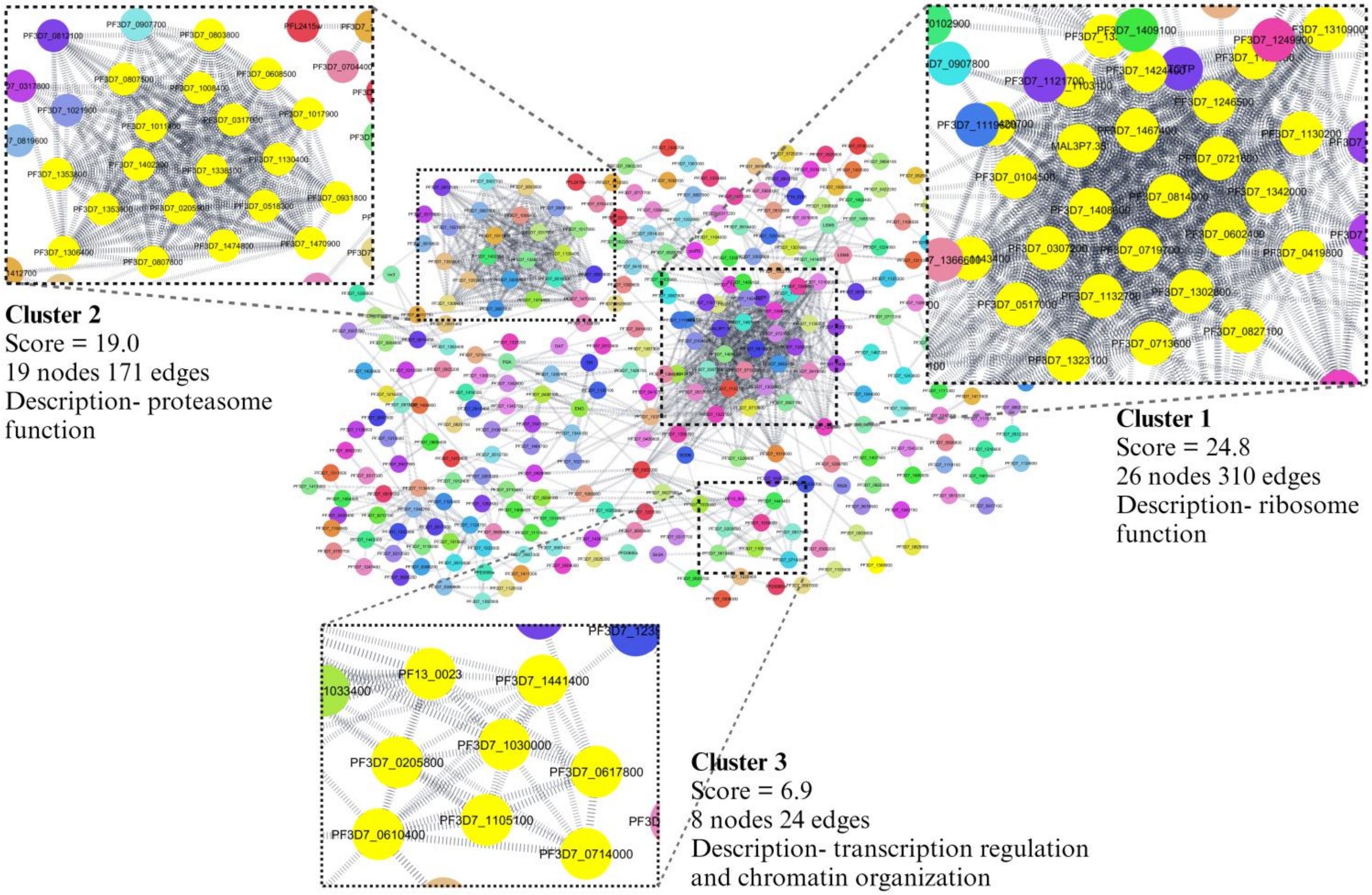

**Figure 5**. **Protein–Protein Interaction Network of *P. falciparum* DEGs in SMA**. Global PPI network generated from *P. falciparum* DEGs identified in SMA, showing the top three of 17 clusters. The network consists of 542 nodes and 1,122 edges (PPI enrichment *p*-value=4.06e-10). Colored circles represent nodes, and connecting lines represent protein interactions. Yellow nodes highlight the top three highly interconnected subnetworks identified by MCODE clustering. Description: Cluster annotations based on enriched functional pathways associated with each subnetwork.

**Table 2. PPI Network Clusters Identified from *P. falciparum* Differentially Expressed Genes**

| Network | Score[a] | Nodes | Edges | Hub Node | Hub Node Description | $Log_2FC$ | *p*-value |
|---|---|---|---|---|---|---|---|
| Global network | | 542 | 1,122 | PF3D7_1331700 | Glutamine--tRNA ligase, putative | 0.612 | 0.016 |
| Cluster 1 | 24.80 | 26 | 310 | PF3D7_1331700 | Glutamine--tRNA ligase, putative | 0.612 | 0.016 |
| Cluster 2 | 19.00 | 19 | 171 | PF3D7_1474800 | Proteasome subunit alpha type-1, putative | 0.454 | 0.044 |
| Cluster 3 | 6.86 | 8 | 24 | PF3D7_0714000 | Histone H2B variant | -0.310 | 0.031 |
| Cluster 4 | 5.60 | 6 | 14 | PF3D7_0512600 | Ras-related protein Rab-1B | 0.474 | 0.027 |
| Cluster 5 | 5.00 | 5 | 10 | PF3D7_0614400 | Pre-mRNA-splicing factor CWF7, putative | 0.619 | 0.035 |
| Cluster 6 | 5.00 | 7 | 15 | PF3D7_1336900 | Tryptophan--tRNA ligase | 0.655 | 0.019 |
| Cluster 7 | 5.00 | 9 | 20 | PF3D7_0816600 | Chaperone protein ClpB1 | 2.361 | 0.022 |
| Cluster 8 | 4.67 | 10 | 21 | PF3D7_0915400 | ATP-dependent 6-phosphofructokinase | 0.519 | 0.030 |
| Cluster 9 | 4.50 | 5 | 9 | PF3D7_1224900 | Splicing factor 3B subunit 6, putative | -0.267 | 0.024 |
| Cluster 10 | 4.00 | 4 | 6 | PF3D7_0409200 | Protein SOF1, putative | -0.224 | 0.045 |
| Cluster 11 | 4.00 | 8 | 14 | PF3D7_0710400 | DNA repair protein RAD14, putative | 0.771 | 0.048 |
| Cluster 12 | 3.33 | 4 | 5 | PF3D7_1409900 | Cytidine diphosphate-diacylglycerol synthase | 1.054 | 0.009 |
| Cluster 13 | 3.00 | 3 | 3 | PF3D7_1015600 | Heat shock protein 60 | 2.063 | 0.032 |
| Cluster 14 | 3.00 | 3 | 3 | PF3D7_1110500 | Vacuolar protein sorting-associated protein 35, putative | 1.228 | 0.045 |
| Cluster 15 | 3.00 | 3 | 3 | PF3D7_1416500 | NADP-specific glutamate dehydrogenase | -0.676 | 0.012 |
| Cluster 16 | 3.00 | 3 | 3 | PF3D7_1237000 | SUMO-activating enzyme subunit 2 | 0.986 | 0.021 |
| Cluster 17 | 2.67 | 4 | 4 | PF3D7_1363400 | Polyubiquitin binding protein, putative | 0.911 | 0.041 |

The table summarizes highly interconnected PPI subnetworks identified by MCODE analysis of *P. falciparum* DEGs. Clusters are ranked according to MCODE scores. Node and edge counts represent cluster membership and protein interactions, respectively. Hub genes for the global PPI network and individual subnetworks were identified using CytoHubba based on 11 topological algorithms. Abbreviation: $Log_2FC$- $Log_2$foldchange. [a] MCODE scores.

**a**

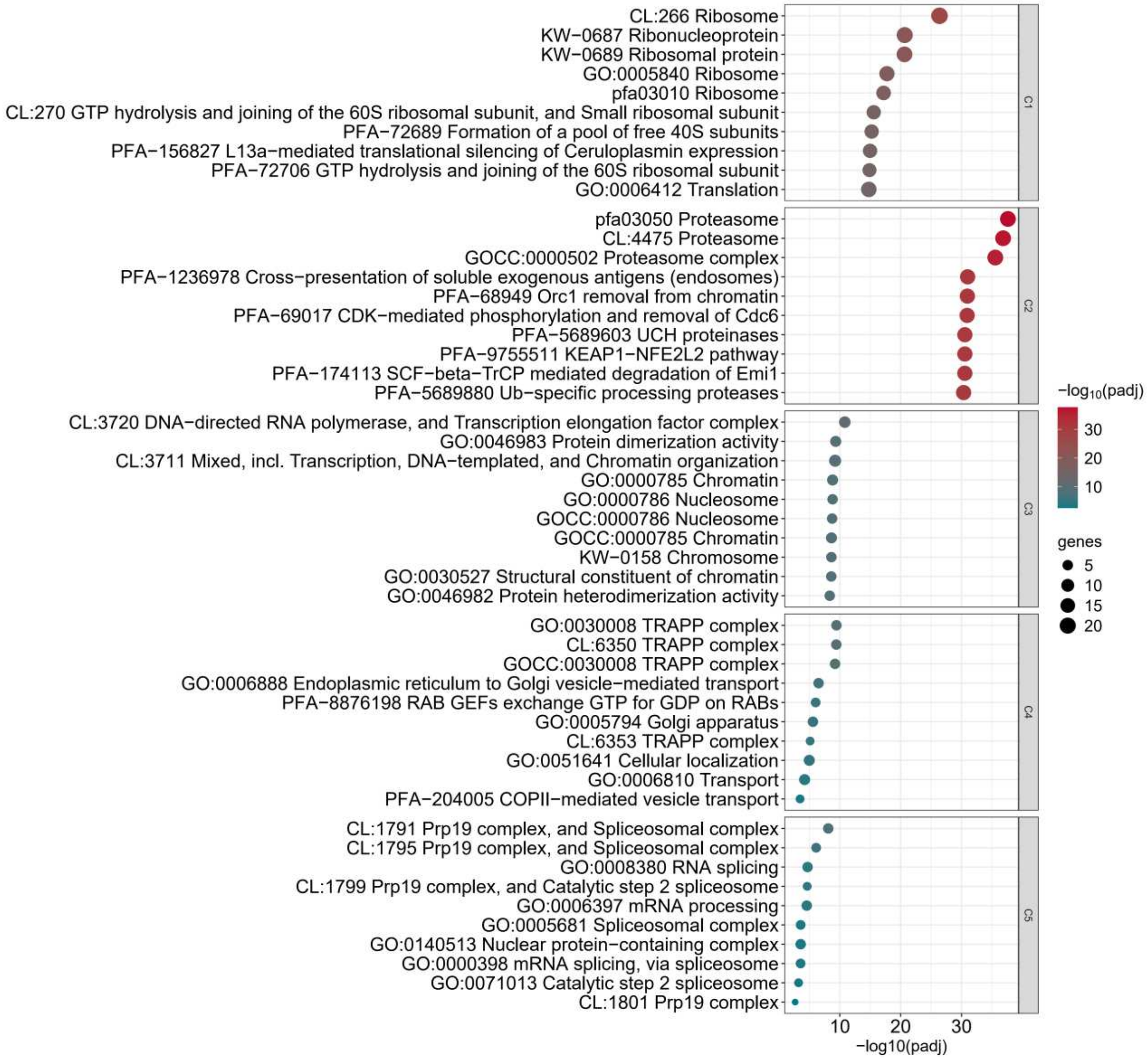

CL:266 Ribosome
KW−0687 Ribonucleoprotein
KW−0689 Ribosomal protein
GO:0005840 Ribosome
pfa03010 Ribosome
CL:270 GTP hydrolysis and joining of the 60S ribosomal subunit, and Small ribosomal subunit
PFA−72689 Formation of a pool of free 40S subunits
PFA−156827 L13a−mediated translational silencing of Ceruloplasmin expression
PFA−72706 GTP hydrolysis and joining of the 60S ribosomal subunit
GO:0006412 Translation
C1
pfa03050 Proteasome
CL:4475 Proteasome
GOCC:0000502 Proteasome complex
PFA−1236978 Cross−presentation of soluble exogenous antigens (endosomes)
PFA−68949 Orc1 removal from chromatin
PFA−69017 CDK−mediated phosphorylation and removal of Cdc6
PFA−5689603 UCH proteinases
PFA−9755511 KEAP1−NFE2L2 pathway
PFA−174113 SCF−beta−TrCP mediated degradation of Emi1
PFA−5689880 Ub−specific processing proteases
C2
CL:3720 DNA−directed RNA polymerase, and Transcription elongation factor complex
GO:0046983 Protein dimerization activity
CL:3711 Mixed, incl. Transcription, DNA−templated, and Chromatin organization
GO:0000785 Chromatin
GO:0000786 Nucleosome
GOCC:0000786 Nucleosome
GOCC:0000785 Chromatin
KW−0158 Chromosome
GO:0030527 Structural constituent of chromatin
GO:0046982 Protein heterodimerization activity
C3
GO:0030008 TRAPP complex
CL:6350 TRAPP complex
GOCC:0030008 TRAPP complex
GO:0006888 Endoplasmic reticulum to Golgi vesicle−mediated transport
PFA−8876198 RAB GEFs exchange GTP for GDP on RABs
GO:0005794 Golgi apparatus
CL:6353 TRAPP complex
GO:0051641 Cellular localization
GO:0006810 Transport
PFA−204005 COPII−mediated vesicle transport
C4
CL:1791 Prp19 complex, and Spliceosomal complex
CL:1795 Prp19 complex, and Spliceosomal complex
GO:0008380 RNA splicing
CL:1799 Prp19 complex, and Catalytic step 2 spliceosome
GO:0006397 mRNA processing
GO:0005681 Spliceosomal complex
GO:0140513 Nuclear protein−containing complex
GO:0000398 mRNA splicing, via spliceosome
GO:0071013 Catalytic step 2 spliceosome
CL:1801 Prp19 complex
C5
10
20
30
−log10(padj)
$-\log_{10}$(padj)
30
20
10
genes
5
10
15
20

**b**

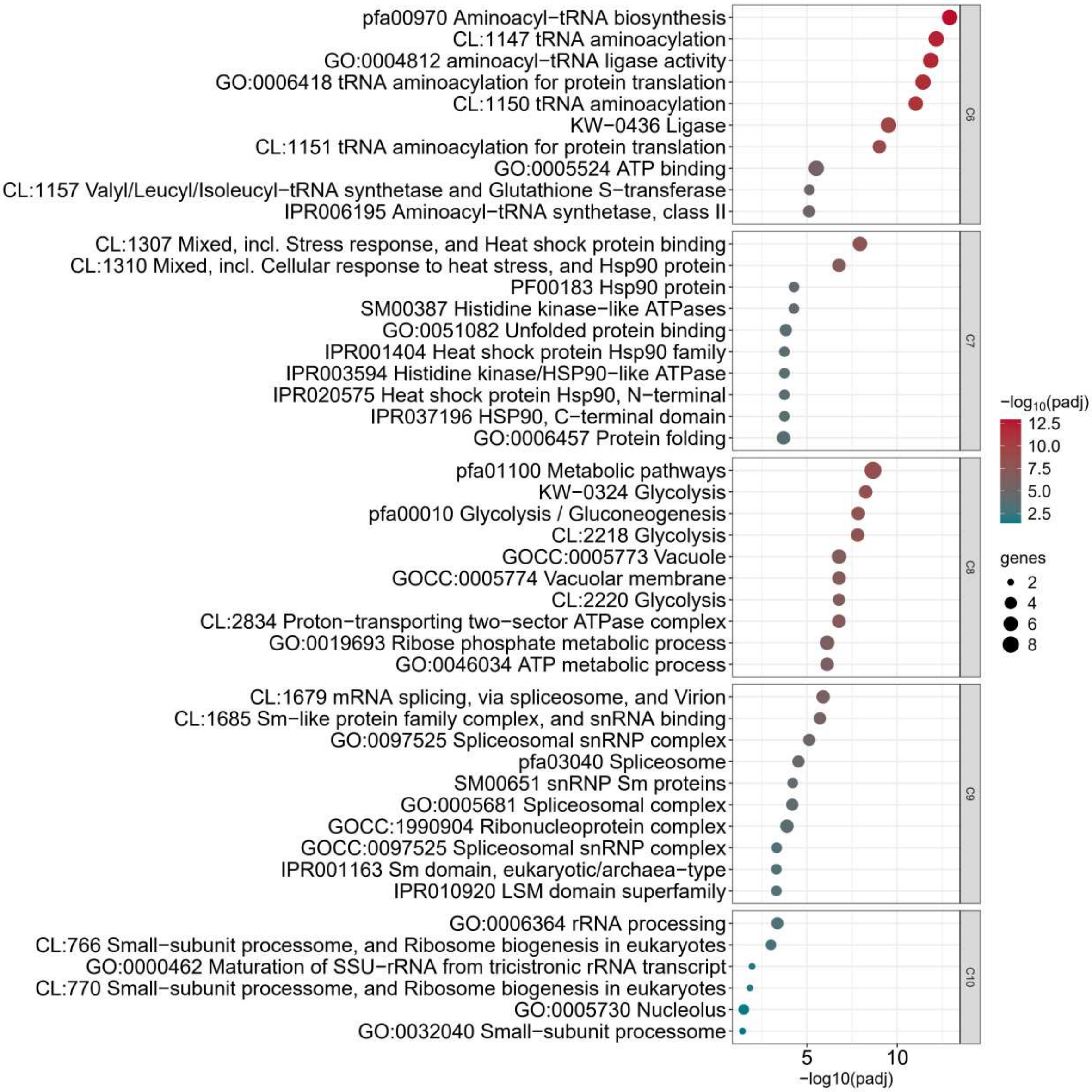

pfa00970 Aminoacyl−tRNA biosynthesis
CL:1147 tRNA aminoacylation
GO:0004812 aminoacyl−tRNA ligase activity
GO:0006418 tRNA aminoacylation for protein translation
CL:1150 tRNA aminoacylation
KW−0436 Ligase
CL:1151 tRNA aminoacylation for protein translation
GO:0005524 ATP binding
CL:1157 Valyl/Leucyl/Isoleucyl−tRNA synthetase and Glutathione S−transferase
IPR006195 Aminoacyl−tRNA synthetase, class II
C6
CL:1307 Mixed, incl. Stress response, and Heat shock protein binding
CL:1310 Mixed, incl. Cellular response to heat stress, and Hsp90 protein
PF00183 Hsp90 protein
SM00387 Histidine kinase−like ATPases
GO:0051082 Unfolded protein binding
IPR001404 Heat shock protein Hsp90 family
IPR003594 Histidine kinase/HSP90−like ATPase
IPR020575 Heat shock protein Hsp90, N−terminal
IPR037196 HSP90, C−terminal domain
GO:0006457 Protein folding
C7
pfa01100 Metabolic pathways
KW−0324 Glycolysis
pfa00010 Glycolysis / Gluconeogenesis
CL:2218 Glycolysis
GOCC:0005773 Vacuole
GOCC:0005774 Vacuolar membrane
CL:2220 Glycolysis
CL:2834 Proton−transporting two−sector ATPase complex
GO:0019693 Ribose phosphate metabolic process
GO:0046034 ATP metabolic process
C8
CL:1679 mRNA splicing, via spliceosome, and Virion
CL:1685 Sm−like protein family complex, and snRNA binding
GO:0097525 Spliceosomal snRNP complex
pfa03040 Spliceosome
SM00651 snRNP Sm proteins
GO:0005681 Spliceosomal complex
GOCC:1990904 Ribonucleoprotein complex
GOCC:0097525 Spliceosomal snRNP complex
IPR001163 Sm domain, eukaryotic/archaea−type
IPR010920 LSM domain superfamily
C9
GO:0006364 rRNA processing
CL:766 Small−subunit processome, and Ribosome biogenesis in eukaryotes
GO:0000462 Maturation of SSU−rRNA from tricistronic rRNA transcript
CL:770 Small−subunit processome, and Ribosome biogenesis in eukaryotes
GO:0005730 Nucleolus
GO:0032040 Small−subunit processome
C10
5
10
−log10(padj)
$-\log_{10}$(padj)
12.5
10.0
7.5
5.0
2.5
genes
2
4
6
8

c

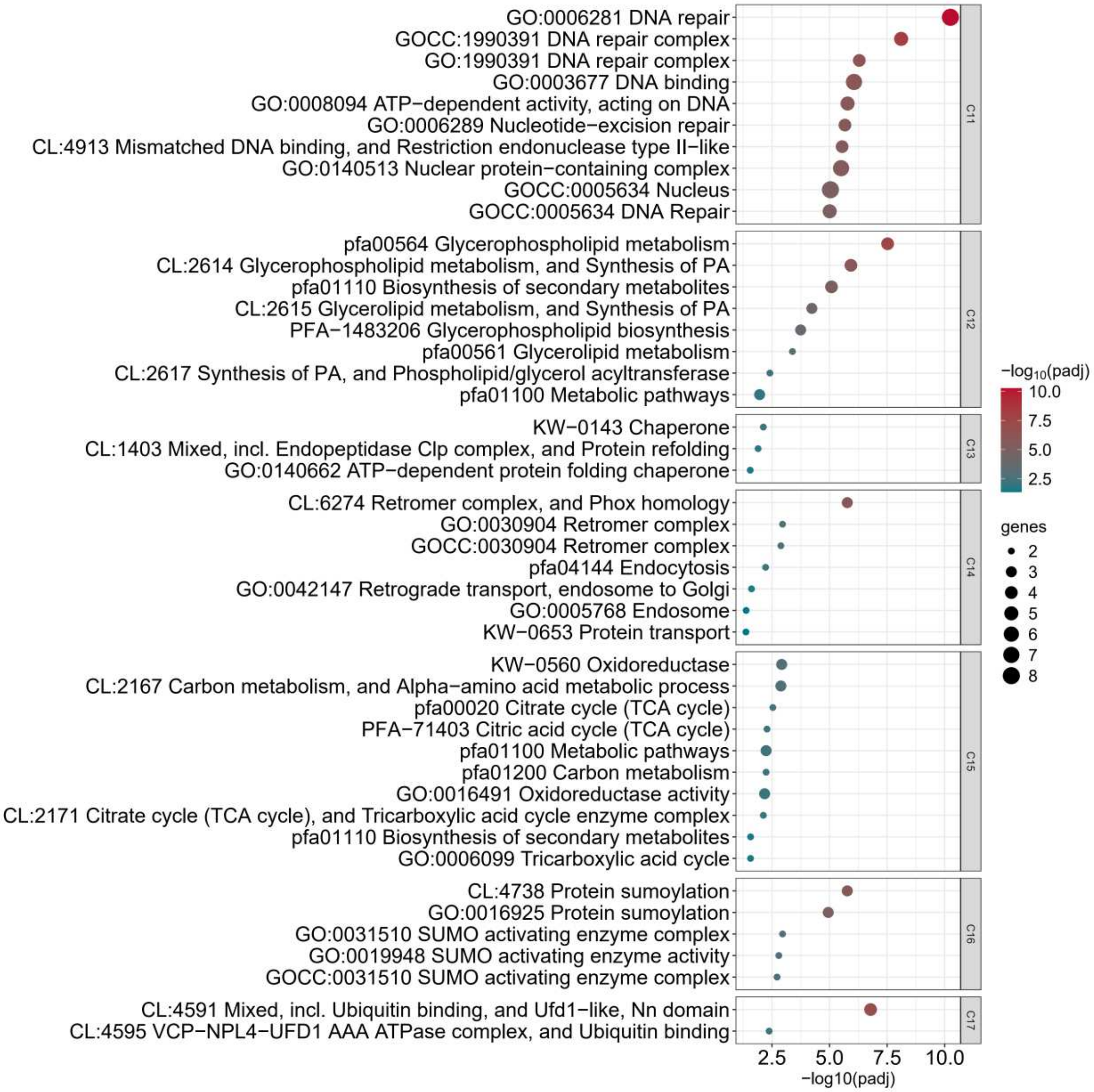

**Figure 6. Functional Enrichment Analysis of *P. falciparum* PPI Subnetworks.** Functional enrichment analysis of *P. falciparum* PPI subnetworks. (**a–c**) Enrichment results for subnetworks 1–5, 6–10, and 11–17, respectively. The x-axis represents –log10(FDR-adjusted *p*-value), and the y-axis shows enriched functional terms. The top 10 most significantly enriched terms are displayed for each subnetwork unless otherwise indicated. Enrichment databases include Gene Ontology (GO), Gene Ontology Cellular Component (GOCC), KEGG *Plasmodium falciparum* annotation (pfa), Reactome *Plasmodium falciparum* annotation (PFA), UniProt Keywords (KW), InterPro Protein Domains (IPR), and STRING cluster databases (CL).

a b

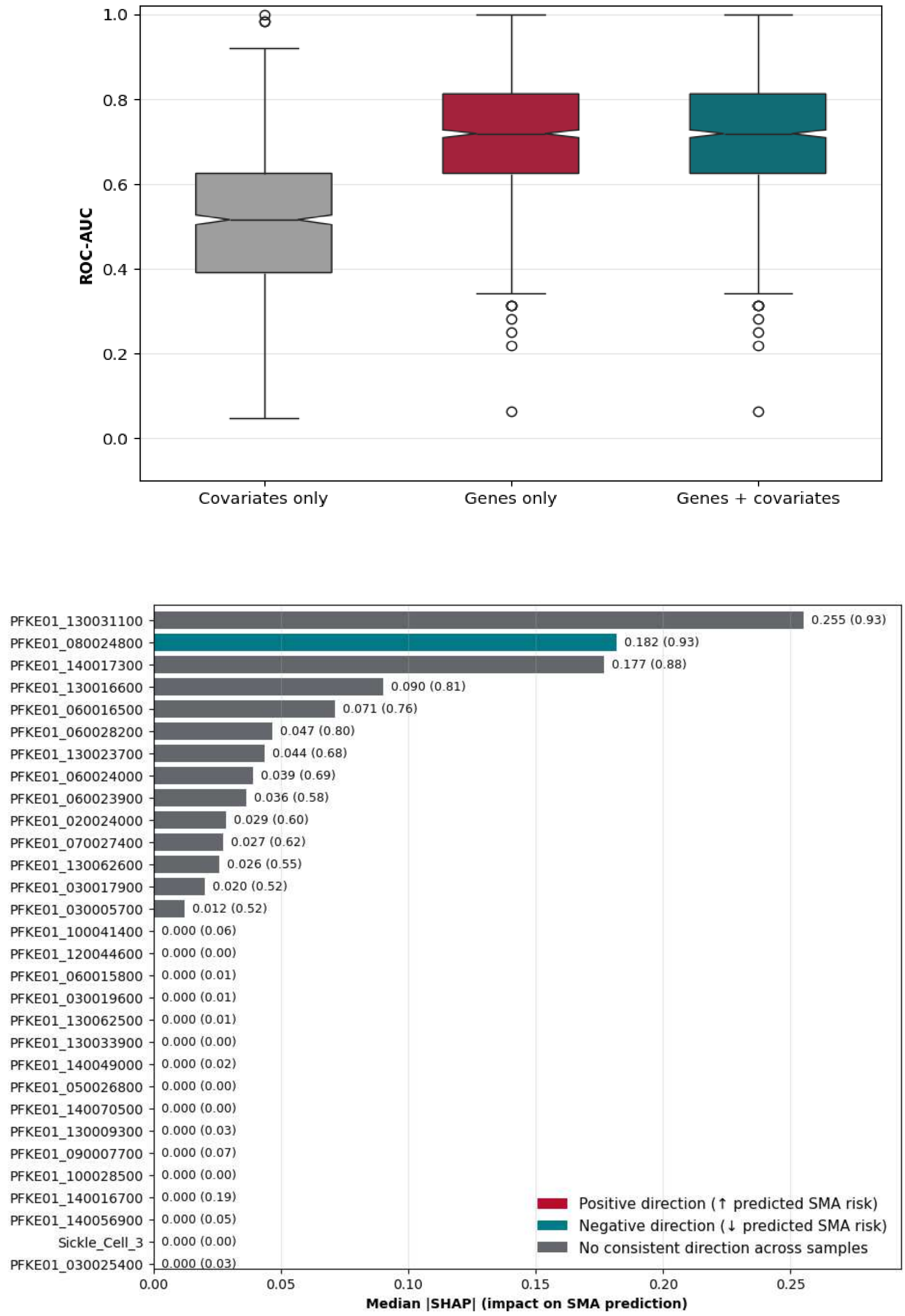

ROC-AUC
1.0
0.8
0.6
0.4
0.2
0.0
Covariates only
Genes only
Genes + covariates
PFKE01_130031100 0.255 (0.93)
PFKE01_080024800 0.182 (0.93)
PFKE01_140017300 0.177 (0.88)
PFKE01_130016600 0.090 (0.81)
PFKE01_060016500 0.071 (0.76)
PFKE01_060028200 0.047 (0.80)
PFKE01_130023700 0.044 (0.68)
PFKE01_060024000 0.039 (0.69)
PFKE01_060023900 0.036 (0.58)
PFKE01_020024000 0.029 (0.60)
PFKE01_070027400 0.027 (0.62)
PFKE01_130062600 0.026 (0.55)
PFKE01_030017900 0.020 (0.52)
PFKE01_030005700 0.012 (0.52)
PFKE01_100041400 0.000 (0.06)
PFKE01_120044600 0.000 (0.00)
PFKE01_060015800 0.000 (0.01)
PFKE01_030019600 0.000 (0.01)
PFKE01_130062500 0.000 (0.01)
PFKE01_130033900 0.000 (0.00)
PFKE01_140049000 0.000 (0.02)
PFKE01_050026800 0.000 (0.00)
PFKE01_140070500 0.000 (0.00)
PFKE01_130009300 0.000 (0.03)
PFKE01_090007700 0.000 (0.07)
PFKE01_100028500 0.000 (0.00)
PFKE01_140016700 0.000 (0.19)
PFKE01_140056900 0.000 (0.05)
Sickle_Cell_3 0.000 (0.00)
PFKE01_030025400 0.000 (0.03)
Positive direction (↑ predicted SMA risk)
Negative direction (↓ predicted SMA risk)
No consistent direction across samples
0.00
0.05
0.10
0.15
0.20
0.25
Median |SHAP| (impact on SMA prediction)

c

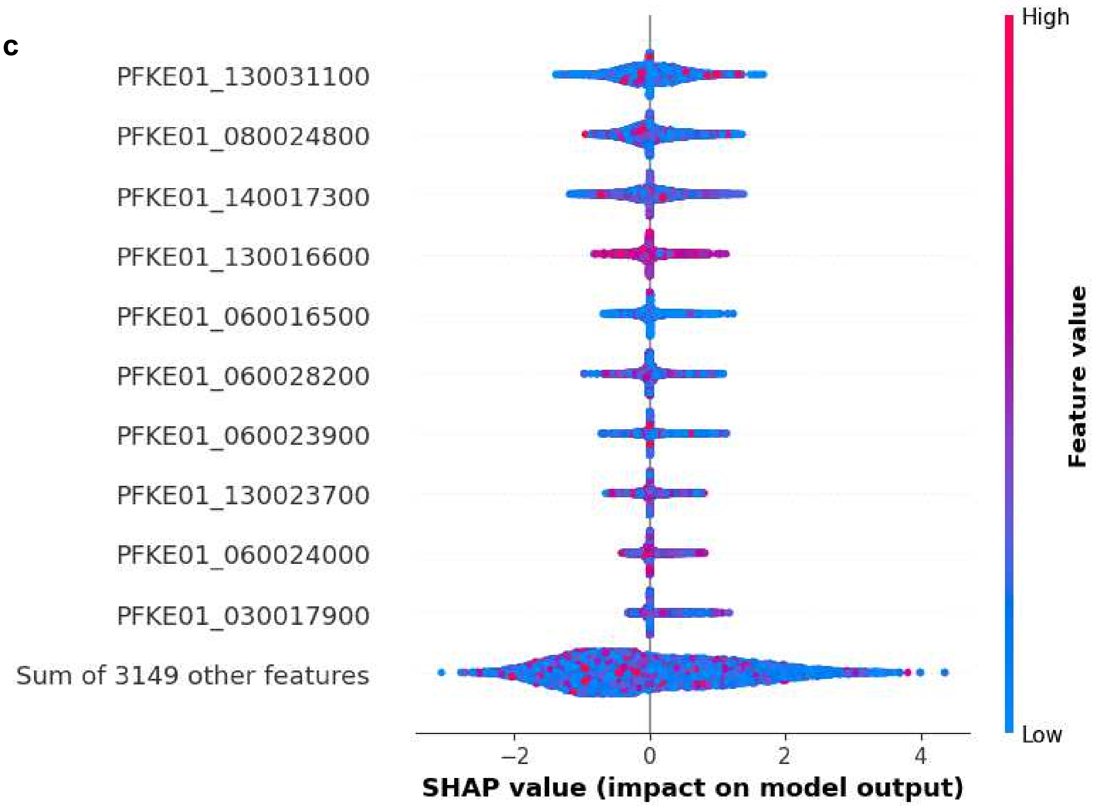

High
PFKE01_130031100
PFKE01_080024800
PFKE01_140017300
PFKE01_130016600
PFKE01_060016500
PFKE01_060028200
PFKE01_060023900
PFKE01_130023700
PFKE01_060024000
PFKE01_030017900
Sum of 3149 other features
Feature value
Low
−2
0
2
4
SHAP value (impact on model output)

d

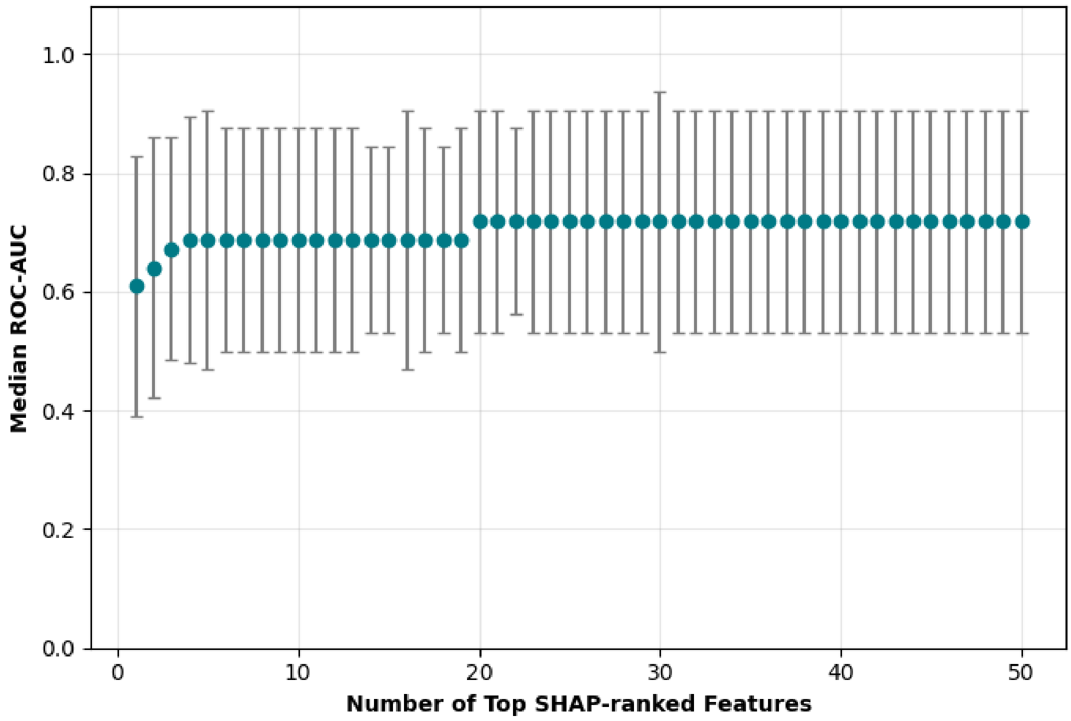

Median ROC-AUC
1.0
0.8
0.6
0.4
0.2
0.0
0
10
20
30
40
50
Number of Top SHAP-ranked Features

**Figure 7. XGBoost and SHAP Analyses Identify Parasite Genes Associated with SMA Classification**. (**a**) Cross-validated performance of XGBoost models classifying SMA. Notched boxplots show median area under the receiver operating characteristic curve (AUC) across stratified 5-fold cross-validation repeated 200 times, with interquartile range (IQR) error bars. (**b**) Directional SHAP feature importance analysis for SMA classification using parasite gene expression profiles and clinical covariates. Features are ranked according to median absolute SHAP values aggregated across repeated cross-validation analyses. Bar color indicates the predominant direction of effect on SMA prediction. Turquoise indicates features associated with decreased predicted SMA risk, whereas gray indicates features without a consistent directional effect across samples. The fraction non-zero indicates the proportion of predictions in which a feature contributed a non-zero SHAP value. Sickle_cell_3: HbSS. (**c**) SHAP beeswarm plot showing sample-level feature contributions to SMA classification. Each point represents a sample-specific SHAP value calculated from held-out test samples during cross-validation. Positive SHAP values increase predicted SMA probability, whereas negative values decrease predicted SMA probability. Point color reflects relative feature expression, with red indicating higher values and blue indicating lower values. Remaining low-impact features are aggregated in the final row. (**d**) Incremental SHAP analysis showing the cumulative contribution of ranked features to SMA classification. Features were sequentially added according to median absolute SHAP importance values derived from repeated stratified cross-validation analyses. The y-axis represents median AUC with interquartile range (IQR) error bars, while the x-axis represents the number of top-ranked features included in the model. Model performance plateaued after including approximately 20 top-ranked features.

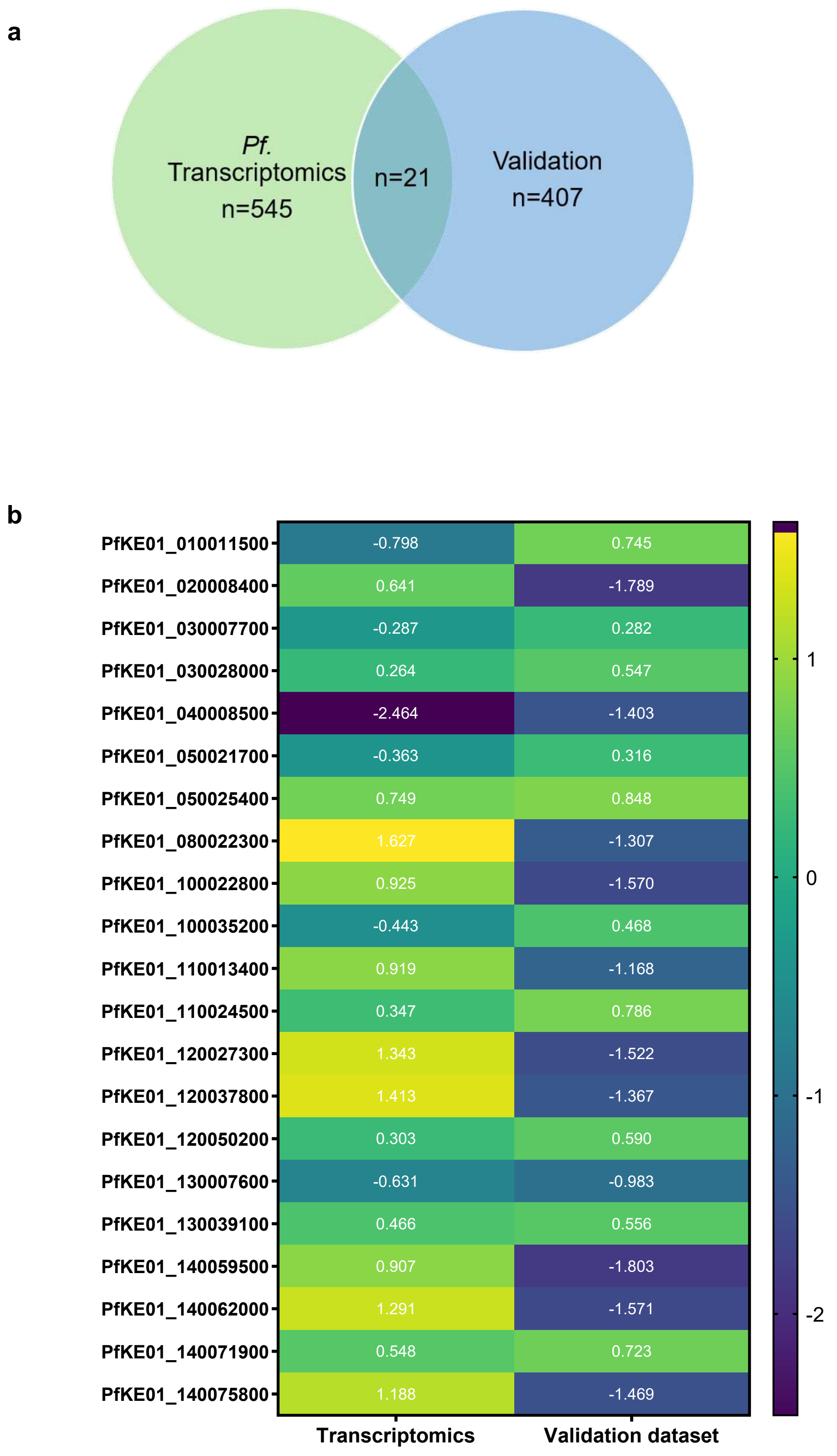

a
Pf.
Transcriptomics
n=545
n=21
Validation
n=407
b
PfKE01_010011500
PfKE01_020008400
PfKE01_030007700
PfKE01_030028000
PfKE01_040008500
PfKE01_050021700
PfKE01_050025400
PfKE01_080022300
PfKE01_100022800
PfKE01_100035200
PfKE01_110013400
PfKE01_110024500
PfKE01_120027300
PfKE01_120037800
PfKE01_120050200
PfKE01_130007600
PfKE01_130039100
PfKE01_140059500
PfKE01_140062000
PfKE01_140071900
PfKE01_140075800
-0.798
0.745
0.641
-1.789
-0.287
0.282
0.264
0.547
-2.464
-1.403
-0.363
0.316
0.749
0.848
1.627
-1.307
0.925
-1.570
-0.443
0.468
0.919
-1.168
0.347
0.786
1.343
-1.522
1.413
-1.367
0.303
0.590
-0.631
-0.983
0.466
0.556
0.907
-1.803
1.291
-1.571
0.548
0.723
1.188
-1.469
Transcriptomics
Validation dataset
1
0
-1
-2

c

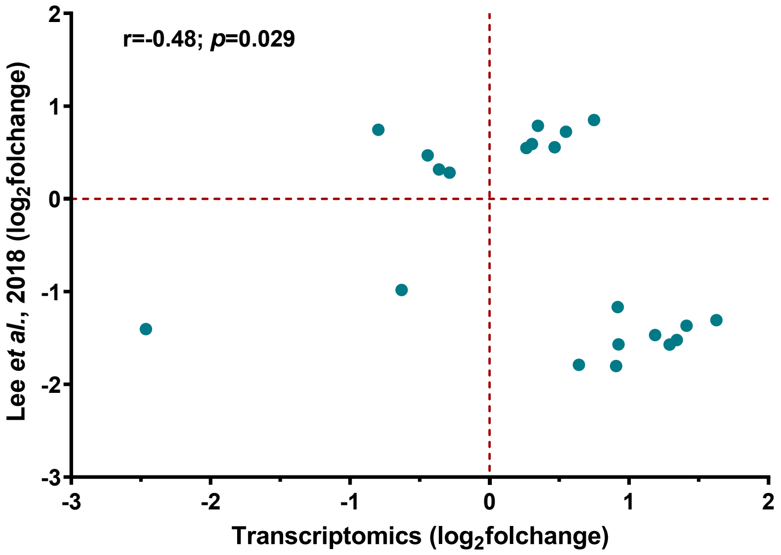

**Figure 8. Independent assessment of the reproducibility of *P. falciparum* parasite transcriptome using an external gene-expression dataset.** (**a**) Venn Diagram depicting the overlap between the DEGs identified in the current study and those identified in the external independent dataset. Unique and shared DEGs within each dataset are shown. (**b**) Heatmap illustrates the magnitude and direction of the intersecting DEGs between the two datasets. The Y-axis depicts the gene pairs, and the X-axis represents the assay type. The color scale depicts fold regulation ($Log_2$). (**c**) Correlation scatter plot showing the relationship between the $log_2$foldchange values for the shared DEGs in the Lee *et al.* 2018 dataset ($log_2$foldchange, Y-axis) versus the current dataset ($log_2$foldchange, X-axis). A significant positive correlation was observed (Spearman $r$=0.491, $p$=2.40e-03).

## Supplementary Files

This is a list of supplementary files associated with this preprint. Click to download.

- Supplementalfiles100226.docx
